# Second asymptomatic carotid surgery trial (ACST-2): a randomised comparison of carotid artery stenting versus carotid endarterectomy

**DOI:** 10.1016/S0140-6736(21)01910-3

**Published:** 2021-09-18

**Authors:** Alison Halliday, Richard Bulbulia, Leo H Bonati, Johanna Chester, Andrea Cradduck-Bamford, Richard Peto, Hongchao Pan, ACST-2 Collaborative Group, Alison Halliday, Richard Bulbulia, Leo H Bonati, Richard Peto, Hongchao Pan, John Potter, Hans Henning Eckstein, Barbara Farrell, Marcus Flather, Averil Mansfield, Boby Mihaylova, Kazim Rahimi, David Simpson, Dafydd Thomas, Peter Sandercock, Richard Gray, Andrew Molyneux, Cliff P Shearman, Peter Rothwell, Anna Belli, Will Herrington, Parminder Judge, Peter Leopold, Marion Mafham, Michael Gough, Piergiorgio Cao, Sumaira MacDonald, Vasha Bari, Clive Berry, S Bradshaw, Wojciech Brudlo, Alison Clarke, Johanna Chester, Robin Cox, Andrea Cradduck-Bamford, Susan Fathers, Kamran Gaba, Mo Gray, Elizabeth Hayter, Constance Holliday, Rijo Kurien, Michael Lay, Steffi le Conte, Jessica McManus, Zahra Madgwick, Dylan Morris, Andrew Munday, Sandra Pickworth, Wiktor Ostasz, Michiel Poorthuis, Sue Richards, Louisa Teixeira, Sergey Tochlin, Lynda Tully, Carol Wallis, Monique Willet, Alan Young, Renato Casana, Chiara Malloggi, Andrea Odero Jr, Vincenzo Silani, Gianfranco Parati, Giuseppe Malchiodi, Giovanni Malferrari, Francesco Strozzi, Nicola Tusini, Enrico Vecchiati, Gioacchino Coppi, Antonio Lauricella, Roberto Moratto, Roberto Silingardi, Jessica Veronesi, Andrea Zini, Emanuele Ferrero, Michelangelo Ferri, Andrea Gaggiano, Carmelo Labate, Franco Nessi, Daniele Psacharopulo, Andrea Viazzo, Giovanni Malacrida, Daniela Mazzaccaro, Giovanni Meola, Alfredo Modafferi, Giovanni Nano, Maria Teresa Occhiuto, Paolo Righini, Silvia Stegher, Stefano Chiarandini, Filippo Griselli, Sandro Lepidi, Fabio Pozzi Mucelli, Marcello Naccarato, Mario D'Oria, Barbara Ziani, Andrea Stella, Mortalla Dieng, Gianluca Faggioli, Mauro Gargiulo, Sergio Palermo, Rodolfo Pini, Giovanni Maria Puddu, Andrea Vacirca, Domenico Angiletta, Claudio Desantis, Davide Marinazzo, Giovanni Mastrangelo, Guido Regina, Raffaele Pulli, Paolo Bianchi, Lea Cireni, Elisabetta Coppi, Rocco Pizzirusso, Filippo Scalise, Giovanni Sorropago, Valerio Tolva, Valeria Caso, Enrico Cieri, Paola DeRango, Luca Farchioni, Giacomo Isernia, Massimo Lenti, Gian Battista Parlani, Guglielmo Pupo, Grazia Pula, Gioele Simonte, Fabio Verzini, Federico Carimati, Maria Luisa Delodovici, Federico Fontana, Gabriele Piffaretti, Matteo Tozzi, Efrem Civilini, Giorgio Poletto, Bernhard Reimers, Barbara Praquin, Sonia Ronchey, Laura Capoccia, Wassim Mansour, Enrico Sbarigia, Francesco Speziale, Pasqualino Sirignano, Danilo Toni, Roberto Galeotti, Vincenzo Gasbarro, Francesco Mascoli, Tiberio Rocca, Elpiniki Tsolaki, Giulia Bernardini, Ester DeMarco, Alessia Giaquinta, Francesco Patti, Massimiliano Veroux, Pierfrancesco Veroux, Carla Virgilio, Nicola Mangialardi, Matteo Orrico, Vincenzo Di Lazzaro, Nunzio Montelione, Francesco Spinelli, Francesco Stilo, Carlo Cernetti, Sandro Irsara, Giuseppe Maccarrone, Diego Tonello, Adriana Visonà, Beniamino Zalunardo, Emiliano Chisci, Stefano Michelagnoli, Nicola Troisi, Maela Masato, Massimo Dei Negri, Andrea Pacchioni, Salvatore Saccà, Giovanni Amatucci, Alfredo Cannizzaro, Federico Accrocca, Cesare Ambrogi, Renzo Barbazza, Giustino Marcucci, Andrea Siani, Guido Bajardi, Giovanni Savettieri, Angelo Argentieri, Riccardo Corbetta, Attilio Odero, Pietro Quaretti, Federico Z Thyrion, Alessandro Cappelli, Domenico Benevento, Gianmarco De Donato, Maria Agnese Mele, Giancarlo Palasciano, Daniela Pieragalli, Alessandro Rossi, Carlo Setacci, Francesco Setacci, Domenico Palombo, Maria Cecilia Perfumo, Edoardo Martelli, Aldo Paolucci, Santi Trimarchi, Viviana Grassi, Luigi Grimaldi, Giuliana La Rosa, Domenico Mirabella, Matteo Scialabba, Leonildo Sichel, Costantino L D'Angelo, Gian Franco Fadda, Holta Kasemi, Mario Marino, Francesco Burzotta, Francesco Alberto Codispoti, Angela Ferrante, Giovanni Tinelli, Yamume Tshomba, Claudio Vincenzoni, Deborah Amis, Dawn Anderson, Martin Catterson, Mike Clarke, Michelle Davis, Anand Dixit, Alexander Dyker, Gary Ford, Ralph Jackson, Sreevalsan Kappadath, David Lambert, Tim Lees, Stephen Louw, James McCaslin, Noala Parr, Rebecca Robson, Gerard Stansby, Lucy Wales, Vera Wealleans, Lesley Wilson, Michael Wyatt, Hardeep Baht, Ibrahim Balogun, Ilse Burger, Tracy Cosier, Linda Cowie, Gunaratnam Gunathilagan, David Hargroves, Robert Insall, Sally Jones, Hannah Rudenko, Natasha Schumacher, Jawaharlal Senaratne, George Thomas, Audrey Thomson, Tom Webb, Ellen Brown, Bernard Esisi, Ali Mehrzad, Shane MacSweeney, Norman McConachie, Alison Southam, Wayne Sunman, Ahmed Abdul-Hamiq, Jenny Bryce, Ian Chetter, Duncan Ettles, Raghuram Lakshminarayan, Kim Mitchelson, Christopher Rhymes, Graham Robinson, Paul Scott, Alison Vickers, Ray Ashleigh, Stephen Butterfield, Ed Gamble, Jonathan Ghosh, Charles N McCollum, Mark Welch, Sarah Welsh, Leszek Wolowczyk, Mary Donnelly, Stephen D'Souza, Anselm A Egun, Bindu Gregary, Thomas Joseph, Christine Kelly, Shuja Punekar, M Asad Rahi, Sonia Raj, Dare Seriki, George Thomson, James Brown, Ragunath Durairajan, Iris Grunwald, Paul Guyler, Paula Harman, Matthew Jakeways, Christopher Khuoge, Ashish Kundu, Thayalini Loganathan, Nisha Menon, Raji O Prabakaran, Devesh Sinha, Vicky Thompson, Sharon Tysoe, Dennis Briley, Chris Darby, Linda Hands, Dominic Howard, Wilhelm Kuker, Ursula Schulz, Rachel Teal, David Barer, Andrew Brown, Susan Crawford, Paul Dunlop, Ramesh Krishnamurthy, Nikhil Majmudar, Duncan Mitchell, Min P Myint, Richard O'Brien, Janice O'Connell, Naweed Sattar, Shanmugam Vetrivel, Jonathan Beard, Trevor Cleveland, Peter Gaines, John Humphreys, Alison Jenkins, Craig King, Daniel Kusuma, Ralph Lindert, Robbie Lonsdale, Raj Nair, Shah Nawaz, Faith Okhuoya, Douglas Turner, Graham Venables, Paul Dorman, Andrea Hughes, Deborah Jones, David Mendelow, Helen Rodgers, Aidas Raudoniitis, Peter Enevoldson, Hans Nahser, Imelda O'Brien, Francesco Torella, Dave Watling, Richard White, Pauline Brown, Dipankar Dutta, Lorraine Emerson, Paula Hilltout, Sachin Kulkarni, Jackie Morrison, Keith Poskitt, Fiona Slim, Sarah Smith, Amanda Tyler, Joanne Waldron, Mark Whyman, Milda Bajoriene, Lucy Baker, Amanda Colston, Bekky Eliot-Jones, Gita Gramizadeh, Catherine Lewis-Clarke, Laura McCafferty, Deborah Oliver, Debbie Palmer, Abhijeet Patil, Suzannah Pegler, Gopi Ramadurai, Aisling Roberts, Tracey Sargent, Shivaprasad Siddegowda, Ravi Singh-Ranger, Akintunde Williams, Lucy Williams, Steve Windebank, Tadas Zuromskis, Lanka Alwis, Jane Angus, Asaipillai Asokanathan, Caroline Fornolles, Diana Hardy, Sophy Hunte, Frances Justin, Duke Phiri, Marie Mitabouana-Kibou, Lakshmanan Sekaran, Sakthivel Sethuraman, Margaret L Tate, Joyce Akyea-Mensah, Stephen Ball, Angela Chrisopoulou, Elizabeth Keene, Alison Phair, Steven Rogers, John V Smyth, Colin Bicknell, Jeremy Chataway, Nicholas Cheshire, Andrew Clifton, Caroline Eley, Richard Gibbs, Mohammad Hamady, Beth Hazel, Alex James, Michael Jenkins, Nyma Khanom, Austin Lacey, Maz Mireskandari, Joanna O'Reilly, Antony Pereira, Tina Sachs, John Wolfe, Ellen Brown, Philip Davey, Gill Rogers, Gemma Smith, Gareth Tervit, Ian Nichol, Andrew Parry, Gavin Young, Simon Ashley, James Barwell, Francis Dix, Azlisham M Nor, Chris Parry, Angela Birt, Paul Davies, Jim George, Anne Graham, Leon Jonker, Thomas Joseph, Nicci Kelsall, Caroline Potts, Toni Wilson, Andrew Clifton, Jamie Crinnion, Larissa Cuenoud, Nikola Aleksic, Srdan Babic, Nenad Ilijevski, Đorde Radak, Dragan Sagic, Slobodan Tanaskovic, Momcilo Colic, Vladimir Cvetic, Lazar Davidovic, Dejana R Jovanovic, Igor Koncar, Perica Mutavdžic, Miloš Sladojevic, Ivan Tomic, Eike S Debus, Ulrich Grzyska, Dagmar Otto, Götz Thomalla, Jessica Barlinn, Johannes Gerber, Kathrin Haase, Christian Hartmann, Stefan Ludwig, Volker Pütz, Christian Reeps, Christine Schmidt, Norbert Weiss, Sebastian Werth, Simon Winzer, Janine Gemper, Albrecht Günther, Bianka Heiling, Elisabeth Jochmann, Panagiota Karvouniari, Carsten Klingner, Thomas Mayer, Julia Schubert, Friederike Schulze-Hartung, Jürgen Zanow, Yvonne Bausback, Franka Borger, Spiridon Botsios, Daniela Branzan, Sven Bräunlich, Henryk Hölzer, Janin Lenzer, Christopher Piorkowski, Nadine Richter, Johannes Schuster, Dierk Scheinert, Andrej Schmidt, Holger Staab, Matthias Ulrich, Martin Werner, Hermann Berger, Gábor Biró, Hans-Henning Eckstein, Michael Kallmayer, Kornelia Kreiser, Alexander Zimmermann, Bärbel Berekoven, Klaus Frerker, Vera Gordon, Giovanni Torsello, Sebastian Arnold, Cora Dienel, Martin Storck, Bernhard Biermaier, Hans Martin Gissler, Christof Klötzsch, Tomas Pfeiffer, Ralph Schneider, Leander Söhl, Michael Wennrich, Angelika Alonso, Michael Keese, Christoph Groden, Andreas Cöster, Andreas Engelhardt, Christoph-Maria Ratusinski, Bengt Berg, Martin Delle, Johan Formgren, Peter Gillgren, Lotta Jarl, Torbjörn B Kall, Peter Konrad, Niklas Nyman, Claes Skiöldebrand, Johnny Steuer, Rabbe Takolander, Jonas Malmstedt, Stefan Acosta, Katarina Björses, Kerstin Brandt, Nuno Dias, Anders Gottsäter, Jan Holst, Thorarinn Kristmundsson, Tobias Kühme, Tilo Kölbel, Bengt Lindblad, Mats Lindh, Martin Malina, Tomas Ohrlander, Tim Resch, Viola Rönnle, Björn Sonesson, Margareta Warvsten, Zbigniew Zdanowski, Erik Campbell, Per Kjellin, Hans Lindgren, Johan Nyberg, Björn Petersen, Gunnar Plate, Håkan Pärsson, Peter Qvarfordt, Pavel Ignatenko, Andrey Karpenko, Vladimir Starodubtsev, Mikhail A Chernyavsky, Maria S Golovkova, Boris B Komakha, Nikolay N Zherdev, Andrey Belyasnik, Pavel Chechulov, Dmitry Kandyba, Igor Stepanishchev, Csaba Csobay-Novák, Edit Dósa, László Entz, Balázs Nemes, Zoltán Szeberin, Pál Barzó, Mihaly Bodosi, Eniko Fákó, Béla Fülöp, Tamás Németh, Szilárd Pazdernyik, Krisztina Skoba, Erika Vörös, Eleni Chatzinikou, Athanasios Giannoukas, Christos Karathanos, Stylianos Koutsias, Georgios Kouvelos, Miltiadis Matsagkas, Styliani Ralli, Christos Rountas, Nikolaos Rousas, Konstantinos Spanos, Elias Brountzos, John D Kakisis, Andreas Lazaris, Konstantinos G Moulakakis, Leonidas Stefanis, Georgios Tsivgoulis, Spyros Vasdekis, Constantine N Antonopoulos, Ion Bellenis, Dimitrios Maras, Antonios Polydorou, Victoria Polydorou, Antonios Tavernarakis, Nikolaos Ioannou, Maria Terzoudi, Miltos Lazarides, Michalis Mantatzis, Kostas Vadikolias, Lukasz Dzieciuchowicz, Marcin Gabriel, Zbigniew Krasinski, Grzegorz Oszkinis, Fryderyk Pukacki, Maciej Slowinski, Michal-Goran Stanišic, Ryszard Staniszewski, Jolanta Tomczak, Maciej Zielinski, Piotr Myrcha, Dorota Rózanski, Stanislaw Drelichowski, Wojciech Iwanowski, Katarzyna Koncewicz, Pawel Bialek, Zbigniew Biejat, Wojciech Czepel, Anna Czlonkowska, Anatol Dowzenko, Julia Jedrzejewska, Adam Kobayashi, Jerzy Leszczynski, Andrzej Malek, Jerzy Polanski, Robert Proczka, Maciej Skorski, Mieczyslaw Szostek, Piotr Andziak, Maciej Dratwicki, Robert Gil, Miroslaw Nowicki, Jaroslaw Pniewski, Jaroslaw Rzezak, Piotr Seweryniak, Pawel Dabek, Michal Juszynski, Grzegorz Madycki, Bartosz Pacewski, Witold Raciborski, Piotr Slowinski, Walerian Staszkiewicz, Martin Bombic, Vladimír Chlouba, Jirí Fiedler, Karel Hes, Petr Koštál, Jindrich Sova, Zdenek Kríž, Mojmír Prívara, Michal Reif, Robert Staffa, Robert Vlachovský, Bohuslav Vojtíšek, Tomáš Hrbác, Martin Kuliha, Václav Procházka, Martin Roubec, David Školoudík, David Netuka, Anna Šteklácová, Vladimír Beneš III, Pavel Buchvald, Ladislav Endrych, Miroslav Šercl, Walter Campos Jr, Ivan B Casella, Nelson de Luccia, André E V Estenssoro, Calógero Presti, Pedro Puech-Leão, Celso R B Neves, Erasmo S da Silva, Cid J Sitrângulo Jr, José A T Monteiro, Gisela Tinone, Marcelo Bellini Dalio, Edwaldo E Joviliano, Octávio M Pontes Neto, Mauricio Serra Ribeiro, Patrick Cras, Jeroen M H Hendriks, Mieke Hoppenbrouwers, Patrick Lauwers, Caroline Loos, Laetitia Yperzeele, Mia Geenens, Dimitri Hemelsoet, Isabelle van Herzeele, Frank Vermassen, Parla Astarci, Frank Hammer, Valérie Lacroix, André Peeters, Robert Verhelst, Silvana Cirelli, Pol Dormal, Annelies Grimonprez, Bart Lambrecht, Philipe Lerut, Eddy Thues, Guy De Koster, Quentin Desiron, Alain Maertens de Noordhout, Danielle Malmendier, Mireille Massoz, Georges Saad, Marc Bosiers, Joren Callaert, Koen Deloose, Estrella Blanco Cañibano, Beatriz García Fresnillo, Mercedes Guerra Requena, Pilar C Morata Barrado, Miguel Muela Méndez, Antonio Yusta Izquierdo, Fernando Aparici Robles, Paula Blanes Orti, Luis García Dominguez, Rafael Martínez López, Manuel Miralles Hernández, José I Tembl Ferrairo, Ángel Chamorro, Juan Macho, Víctor Obach, Vincent Riambau, Luis San Román, Frank J Ahlhelm, Kristine Blackham, Stefan Engelter, Thomas Eugster, Henrik Gensicke, Lorenz Gürke, Philippe Lyrer, Luigi Mariani, Marina Maurer, Edin Mujagic, Mandy Müller, Marios Psychogios, Peter Stierli, Christoph Stippich, Christopher Traenka, Thomas Wolff, Benjamin Wagner, Martina M Wiegert, Sandra Clarke, Michael Diepers, Ernst Gröchenig, Lorenz Gürke, Philipp Gruber, Andrej Isaak, Timo Kahles, Regula Marti, Krassen Nedeltchev, Luca Remonda, Peter Stierli, Nadir Tissira, Martina Valença Falcão, Gert J de Borst, Rob H Lo, Frans L Moll, Raechel Toorop, Bart H van der Worp, Evert J Vonken, Jaap L Kappelle, Ommid Jahrome, Floris Vos, Wouter Schuiling, Hendrik van Overhagen, Rudolf W M Keunen, Bob Knippenberg, Jan J Wever, Jan W Lardenoije, Michel Reijnen, Luuk Smeets, Steven van Sterkenburg, Gustav Fraedrich, Elke Gizewski, Ingrid Gruber, Michael Knoflach, Stefan Kiechl, Barbara Rantner, Timur Abdulamit, Patrice Bergeron, Raymond Padovani, Jean-Christophe Trastour, Jean-Marie Cardon, Anne Le Gallou-Wittenberg, Eric Allaire, Jean-Pierre Becquemin, Frédéric Cochennec-Paliwoda, Pascal Desgranges, Hassan Hosseini, Hicham Kobeiter, Jean Marzelle, Mohammed A Almekhlafi, Simerpreet Bal, Phillip A Barber, Shelagh B Coutts, Andrew M Demchuk, Muneer Eesa, Michelle Gillies, Mayank Goyal, Michael D Hill, Mark E Hudon, Anitha Jambula, Carol Kenney, Gary Klein, Marie McClelland, Alim Mitha, Bijoy K Menon, William F Morrish, Steven Peters, Karla J Ryckborst, Greg Samis, Supriya Save, Eric E Smith, Peter Stys, Suresh Subramaniam, Garnette R Sutherland, Tim Watson, John H Wong, L Zimmel, Vojko Flis, Jože Matela, Kazimir Miksic, Franko Milotic, Božidar Mrdja, Barbara Stirn, Erih Tetickovic, Mladen Gasparini, Anton Grad, Ingrid Kompara, Zoren Miloševic, Veronika Palmiste, Toomas Toomsoo, Balzhan Aidashova, Nursultan Kospanov, Roman Lyssenko, Daulet Mussagaliev, Rafi Beyar, Aaron Hoffman, Tony Karram, Arthur Kerner, Eugenia Nikolsky, Samy Nitecki, Silva Andonova, Chavdar Bachvarov, Vesko Petrov, Ivan Cvjetko, Vinko Vidjak, Damir Halužan, Mladen Petrunic, Bao Liu, Chang-Wei Liu, Daniel Bartko, Peter Beno, František Rusnák, Kamil Zelenák, Masayuki Ezura, Takashi Inoue, Naoto Kimura, Ryushi Kondo, Yasushi Matsumoto, Hiroaki Shimizu, Hidenori Endo, Eisuke Furui, Søren Bakke, Kristen Krohg-Sørensen, Terje Nome, Mona Skjelland, Bjørn Tennøe, João Albuquerque e Castro, Gonçalo Alves, Frederico Bastos Gonçalves, José de Aragão Morais, Ana C Garcia, Hugo Valentim, Leonor Vasconcelos, Fernando Belcastro, Fernando Cura, Patricio Zaefferer, Foad Abd-Allah, Mohamed H Eldessoki, Hussein Heshmat Kassem, Haytham Soliman Gharieb, Mary P Colgan, Syed N Haider, Joe Harbison, Prakash Madhavan, Dermot Moore, Gregor Shanik, Viviane Kazan, Munier Nazzal, Vicki Ramsey-Williams

**Affiliations:** a Nuffield Department of Population Health, University of Oxford, Oxford, UK; b Nuffield Department of Surgery, University of Oxford, Oxford, UK; c Department of Neurology, University Hospital, Basel, Switzerland; d Medical Research Council Population Health Research Unit, Nuffield Department of Population Health, University of Oxford, Oxford, UK

## Abstract

**Background:**

Among asymptomatic patients with severe carotid artery stenosis but no recent stroke or transient cerebral ischaemia, either carotid artery stenting (CAS) or carotid endarterectomy (CEA) can restore patency and reduce long-term stroke risks. However, from recent national registry data, each option causes about 1% procedural risk of disabling stroke or death. Comparison of their long-term protective effects requires large-scale randomised evidence.

**Methods:**

ACST-2 is an international multicentre randomised trial of CAS versus CEA among asymptomatic patients with severe stenosis thought to require intervention, interpreted with all other relevant trials. Patients were eligible if they had severe unilateral or bilateral carotid artery stenosis and both doctor and patient agreed that a carotid procedure should be undertaken, but they were substantially uncertain which one to choose. Patients were randomly allocated to CAS or CEA and followed up at 1 month and then annually, for a mean 5 years. Procedural events were those within 30 days of the intervention. Intention-to-treat analyses are provided. Analyses including procedural hazards use tabular methods. Analyses and meta-analyses of non-procedural strokes use Kaplan-Meier and log-rank methods. The trial is registered with the ISRCTN registry, ISRCTN21144362.

**Findings:**

Between Jan 15, 2008, and Dec 31, 2020, 3625 patients in 130 centres were randomly allocated, 1811 to CAS and 1814 to CEA, with good compliance, good medical therapy and a mean 5 years of follow-up. Overall, 1% had disabling stroke or death procedurally (15 allocated to CAS and 18 to CEA) and 2% had non-disabling procedural stroke (48 allocated to CAS and 29 to CEA). Kaplan-Meier estimates of 5-year non-procedural stroke were 2·5% in each group for fatal or disabling stroke, and 5·3% with CAS versus 4·5% with CEA for any stroke (rate ratio [RR] 1·16, 95% CI 0·86–1·57; p=0·33). Combining RRs for any non-procedural stroke in all CAS versus CEA trials, the RR was similar in symptomatic and asymptomatic patients (overall RR 1·11, 95% CI 0·91–1·32; p=0·21).

**Interpretation:**

Serious complications are similarly uncommon after competent CAS and CEA, and the long-term effects of these two carotid artery procedures on fatal or disabling stroke are comparable.

**Funding:**

UK Medical Research Council and Health Technology Assessment Programme.

## Introduction

Severely stenosed carotid arteries predispose to stroke, and either carotid artery stenting (CAS) or carotid endarterectomy (CEA) can restore patency and reduce the long-term risk of stroke. Open carotid artery surgery completely removes the atheromatous material, but stenting is less invasive. In North America, some 100 000 surgery or stenting procedures are done each year to treat carotid artery narrowing,^1^ and numbers are similar for Europe.^2^, ^3^ About half are to prevent recurrent stroke in symptomatic patients and half are for primary stroke prevention in asymptomatic patients (ie, those whose stenosis has not caused any recent ipsilateral symptoms), but this proportion varies from one country to another.^2^ Among asymptomatic patients with severe (eg, 70–99%) stenosis, successful CEA approximately halves the long-term stroke risk.^4^, ^5^

Both CAS and CEA, however, carry a short-term risk of stroke, which is about twice as great for symptomatic as for asymptomatic patients.^3^ When carotid procedures first became common, these risks were substantial, but nowadays they are much lower, particularly among asymptomatic patients. In Germany, for example, where all carotid procedures must, by law, be registered, during 2014–19, the in-hospital risk of disabling stroke or death among asymptomatic patients undergoing CAS (n=18 000) or CEA (n=86 000) was 0·7% for each procedure (appendix p 9); the additional in-hospital risk of non-disabling stroke was 1·1% for CAS and 0·7% for CEA. These rates are below the conventional 3% safety threshold, although only about two thirds of procedural strokes occur before hospital discharge. In this large German registry, the in-hospital risk of stroke after a carotid procedure was reliably shown to be unrelated to age or sex.^3^ In-hospital mortality from other causes was similar in both sexes but increased with age to nearly 1% after age 80 years.^6^

**Research in context**

**Evidence before this study**

In patients with severe carotid artery stenosis, carotid artery stenting (CAS) and carotid endarterectomy (CEA) both carry procedural risks, which are about twice as great for symptomatic as for asymptomatic patients, but they can restore patency and approximately halve long-term stroke rates in asymptomatic patients. The procedural risks have decreased over the decades, but there is still about a 1% risk of disabling stroke or death. There is also some procedural risk of non-disabling stroke (particularly with CAS) or of non-fatal myocardial infarction or cranial nerve palsy (particularly with CEA). Modern drug therapy can also reduce stroke rates but even with it, patients with severe carotid stenosis might have a risk of about 1% per year of disabling stroke or death. Hence, in addition to good medical therapy, carotid procedures are still considered appropriate for many patients. However, there is often uncertainty as to whether CAS or CEA would be more appropriate. Previous trials, first among symptomatic and then among asymptomatic patients, have directly compared CAS versus CEA. Particularly for asymptomatic patients, however, the numbers randomised have been limited, as shown by the 2020 Cochrane review led by LHB (which defines the search strategy for such trials used in the present report). The aim of this study was to randomly assign substantial numbers of asymptomatic patients, and then to consider the results in the context of those from all other trials of CAS versus CEA.

**Added value of this study**

ACST-2 has randomly allocated 3625 asymptomatic patients with severe carotid stenosis to CAS or CEA with good compliance and, thus far, a mean of 5 years of follow-up. The procedures themselves each involved a 1% risk of causing disabling stroke or death but, after each of them, the annual rate of disabling or fatal stroke was only about 0·5%. This study has more than doubled the number of asymptomatic patients in trials of CAS versus CEA. However, the randomised evidence from both asymptomatic and symptomatic patients is relevant to any comparison between the two procedures. With ACST-2 included, there is now as much evidence among asymptomatic as among symptomatic patients, and the findings in both types of patient are remarkably similar, with CAS about as effective as CEA at reducing the annual risk of stroke, at least for the first few years.

**Implications of all the available evidence**

The trials of CAS versus CEA now provide better evidence than existed before that both procedures carry similar risks and provide comparable benefits. This does not address the question of whether, in addition to good medical therapy, a skilful carotid intervention would be appropriate, nor does it address the question of how much each procedure costs to health services or patients. It does, however, mean that doctors and patients have a freer choice of which procedure is more appropriate for individuals.

The current hazards of these two procedures may be better estimated by evidence from large, representative, up-to-date registries than by evidence from randomised trials. What trials can achieve, however (and analyses of registries or other health-care databases cannot),^7^ is a reliably unbiased comparison between the long-term protective effects of CAS and CEA. Although improvements in medical treatment in recent decades have reduced the absolute stroke rates after CAS and after CEA, the relative risks in large trials of them can still determine whether there are any real differences in efficacy.

The evidence thus far from randomised trials of CAS versus CEA suggests approximate similarity of the long-term protective effects of the two procedures, but it has involved only limited numbers of asymptomatic patients.^8^, ^9^ The ACST-2 trial, with a larger number of participants, aimed to provide more robust comparisons of the long-term protective effects of CAS versus CEA.

## Methods

### Study design and participants

ACST-2 is an international multicentre randomised trial done in 33 countries (appendix pp 3–8). Asymptomatic patients with carotid artery stenosis who were thought suitable for CAS or for CEA could enter ACST-2 if the doctor and patient were both substantially uncertain which procedure to prefer. All other aspects of the management of patients were left to the discretion of the clinician and usually included antithrombotic, antihypertensive, and lipid-lowering therapy. The trial compared the 30-day hazards of the two procedures when done by experienced doctors and the subsequent stroke rates over the following 5–10 years. The original trial protocol is provided in the appendix (pp 19–46).

130 hospitals collaborated, each with a vascular surgeon, an interventionalist (perhaps the same person), and a neurologist (or stroke doctor). Potential collaborators submitted a record of their CAS or CEA experience and procedural outcomes. These were anonymised and reviewed; for participation, the risks of any stroke or death had to be 6% or lower for symptomatic patients and 3% or lower for asymptomatic patients. Ethics approval was obtained at each centre and at the UK coordinating centre. Written informed consent was given before randomisation. Interim analyses were supplied annually to the Data Monitoring Committee but never justified disclosure.

Carotid artery stenosis, generally rounded to 60%, 70%, 80%, or 90% (table 1), was assessed by duplex Doppler using locally validated criteria, which would have varied somewhat from one centre to another. In about half the patients, plaque echolucency was also estimated. No images were collected centrally.

**Table 1:** Characteristics of the 3625 patients

	**Allocated CAS (n=1811)**	**Allocated CEA (n=1814)**
**Sex**		
Male	1272 (70%)	1273 (70%)
Female	539 (30%)	541 (30%)
**Age, years**		
<70	909 (50%)	893 (49%)
≥70	902 (50%)	921 (51%)
**Total cholesterol, mmol/L**		
<4·5	601 (33%)	612 (34%)
≥4·5	682 (38%)	708 (39%)
Not measured	528 (29%)	494 (27%)
**HDL cholesterol, mmol/L**		
<1	227 (13%)	263 (14%)
≥1	968 (53%)	966 (53%)
Not measured	616 (34%)	585 (32%)
**Systolic blood pressure, mm Hg**		
≤140	1143 (63%)	1156 (64%)
>140	668 (37%)	658 (36%)
**Medical history**		
Atrial fibrillation	112 (6%)	112 (6%)
Diabetes	542 (30%)	543 (30%)
Coronary artery disease	659 (36%)	642 (35%)
Renal impairment	162 (9%)	145 (8%)
**Previous brain infarct (CT or MRI)**		
Yes	355 (20%)	310 (17%)
No	1115 (62%)	1137 (63%)
Not done	341 (19%)	367 (20%)
**Previous carotid territory symptoms**		
Contralateral	272 (15%)	257 (14%)
Ipsilateral (not in preceding 6 months)	129 (7%)	106 (6%)
**Contralateral carotid stenosis, %**		
<50	1102 (61%)	1104 (61%)
50–99	576 (32%)	585 (32%)
100	133 (7%)	125 (7%)
**Ipsilateral carotid stenosis, %**		
<70 (mean 61)	60 (3%)	58 (3%)
70–79 (mean 72)	631 (35%)	630 (35%)
80–89 (mean 81)	702 (39%)	706 (39%)
90–99 (mean 91)	418 (23%)	420 (23%)
**Ipsilateral plaque echolucency, %**		
<25	592 (33%)	594 (33%)
≥25	546 (30%)	548 (30%)
Not estimated	673 (37%)	672 (37%)
**Medication reported at entry**		
Antiplatelet	1644 (91%)	1624 (90%)
Anticoagulant	148 (8%)	158 (9%)
Antihypertensive	1585 (88%)	1580 (87%)
Lipid-lowering	1530 (84%)	1539 (85%)

Data are n (%). CAS=carotid artery surgery. CEA=carotid endarterectomy.

Patients were eligible if they had severe unilateral or bilateral carotid artery stenosis (generally 60% or higher on ultrasound); this had not caused any relevant neurological symptoms in the preceding 6 months; there was CT or MRI confirmation of suitability for CAS and for CEA (which would also have been used to exclude from trial entry any patient without sufficient stenosis to justify intervention); the doctor and patient agreed that a carotid procedure should be undertaken, but they were substantially uncertain whether this should be CAS or CEA; and the patient had no known circumstance or condition likely to preclude long-term follow-up.

Exclusion criteria included previous ipsilateral intervention, unsuitability for CAS (eg, due to calcification or tortuosity) or CEA, high procedural risk (eg, because of recent acute myocardial infarction), high risk of cardiac emboli, or any major life-threatening condition. Patients likely to require other surgery could not enter the trial until at least 1 month after it.

### Randomisation

Informed consent was obtained before randomisation. Electronic entry through the Oxford Clinical Trial Service Unit recorded patient characteristics before a computer generated the 1:1 random allocation to ipsilateral CAS or CEA. The allocation was minimised on patient characteristics but, to avoid local foreknowledge,^10^ not on centre. Anonymised clinical records were reviewed by the Endpoint Committee, after masking any information indicating the allocated or actual treatment. Masking was complete for non-procedural events, but for procedural events (ie, those occurring before 30 days after the intervention) it was sometimes not possible.

### Procedures

Collaborators used their normal procedures. For CAS, any CE-approved devices were allowed, and procedural double antiplatelet therapy was usual. For CEA, shunting and patching were optional. Long-term medical care was to be similar in both groups and generally involved antithrombotic, antihypertensive, and lipid-lowering therapy.

No tests for silent myocardial infarction were required. Patients were assessed neurologically after their procedure by the collaborating neurologist or stroke doctor, either while still in hospital or within 30 days. Follow-up reports were at 1 month after treatment (including procedural morbidity and duplex ultrasound), and yearly after randomisation (reporting on drug treatment, any later carotid procedures, and any strokes or deaths). Follow-up is continuing (for up to 12 years, thus far). UK death certificates were sent automatically to the trial office; elsewhere, mortality follow-up was through collaborating hospitals or the annual enquiries to patients or carers. If probable strokes were reported, neurological assessments were sought (preferably including CT or MRI).

### Outcomes and endpoint classification

The main trial outcomes were procedural mortality and morbidity (ie, onset before 30 days after the intervention) and, most importantly, non-procedural stroke, subdivided by severity. Strokes had to involve symptoms lasting more than 24 h; any imaging was used to help define the nature of the stroke. Confirmed strokes were classified by site, nature, and eventual outcome after 6 months: non-disabling (modified Rankin Scale [mRS] score 0–2, which involves at most slight residual disability because of the stroke, with patients still able to walk and look after their own affairs without assistance), disabling (mRS score 3–5, which involves at least moderate disability from the stroke, with patients requiring help), or fatal (causing death in any direct or indirect way, regardless of the time between stroke onset and death). The mRS scores are defined more fully in the study protocol (appendix p 26). If the patient died of another cause within 6 months of stroke onset, an estimate of stroke severity was made. A fatal stroke was one that caused death, either directly or indirectly, regardless of the delay between stroke and death; thus, procedural strokes could take more than 30 days to prove fatal. Confirmation of a myocardial infarction required at least two of three criteria: symptoms, biomarker elevation, or electrocardiogram changes.

### Statistical analysis

The original intent was to randomly assign 1000 patients per year for 5 years between CAS and CEA, then follow up all for 5 years after the last patient entered. To facilitate rapid recruitment, entry procedures were simplified and the consent form had a simple front and back page (with details elsewhere only for those wanting them). If, however, prospective participants had already been referred for stenting or for CEA, eligibility for randomisation required consideration of an alternative procedure. Hence, only about 300 patients per year were randomly assigned between CAS and CEA and, after 5 years, investigators were invited to continue randomising, aiming for a reduced target of 3600. The protocol was not modified, as collaboration with other trials was arranged that would yield in total more than 5000 patients by 2020.

The only written statistical analysis plan was that in the protocol (appendix pp 19–46), where the stated primary objectives were to compare the effects of CAS and CEA on procedural risks and on “long-term (up to 5 or more years) prevention of stroke, particularly disabling or fatal stroke”. The sample size considerations imply that the results from ACST-2 should be analysed not in isolation but in conjunction with those from other trials. Analyses in the Discussion include all trials of CAS versus CEA found by the literature-searching strategy of the 2020 Cochrane review (which was led by LHB).

Procedural hazards in nationally representative large registries are now available. Hence, the most important trial results are those on non-procedural strokes. When combining such results from several trials, inverse-variance-weighted averages of the log of the rate ratio (RR) in each trial were used, with 95% CIs for the overall result and 99% confidence limits for each separate trial result. All p values are two-sided.

Analyses of procedural risks related to the first intervention after randomisation, and the intention-to-treat (ITT) analyses were complemented by analyses of procedural risks in those who actually underwent CAS or CEA. The main analyses of non-procedural stroke rates involved log-rank methods. Follow-up was to death or last report of being alive.

ITT analyses and Kaplan-Meier time-to-first-event graphs are provided for all outcomes. Standard continuity-corrected methods for 2 × 2 tables are used for p value calculation for any outcomes that include procedural hazards. Proportional hazards methods (log-rank tests, stratified by age [younger than 65 years, 65–74 years, and 75 years or older] and sex into six groups) are used for p value calculation for non-procedural stroke RRs.

For non-procedural stroke rates, the log of the event RR is estimated from the log-rank observed minus expected (O–E) and its variance V as (O–E)/V, taken to be normally distributed with variance 1/V. This leads to χ^2^ tests of interaction between various baseline features and the effects of treatment allocation on non-procedural stroke rates. Summation of these χ^2^ tests (and, separately, of their degrees of freedom) leads to an approximate global χ^2^ test of the relevance of any of these features to the trial treatment comparison.

Proportional-hazard methods are not used for analyses that combine procedural hazards with long-term stroke rates. For early risk from an intervention may be followed by later benefit, so the hazard ratio comparing one treatment versus another could well go first in one direction then in another (invalidating methods that assume approximately constant hazard ratios). Analyses used SAS, version 9.4, and R, version 4.1. The trial is registered with the ISRCTN registry, ISRCTN21144362.

### Role of the funding source

The study sponsors had no role in design, data collection, analysis, interpretation, or report writing.

## Results

**Figure 1:**
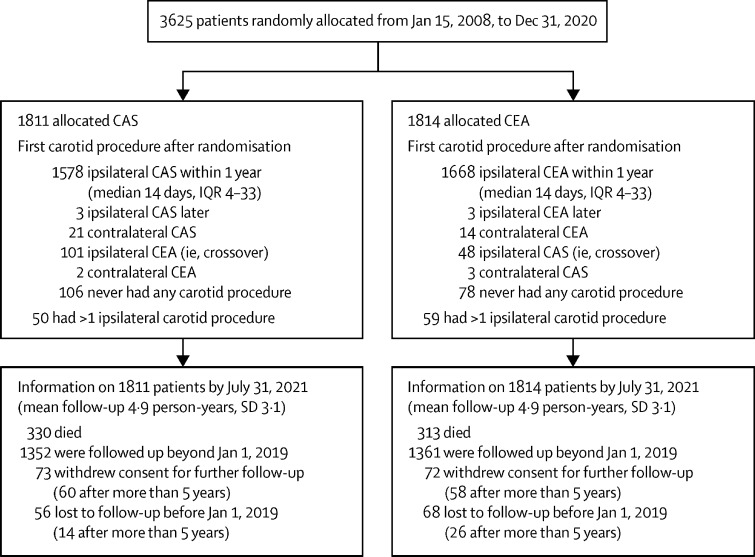
Trial profile CAS=carotid artery stenting. CEA=carotid endarterectomy.

3625 patients from 130 centres in 33 countries were randomly allocated between Jan 15, 2008, and Dec 31, 2020, 1811 to CAS and 1814 to CEA. As minimised randomisation was used, patient characteristics did not differ (table 1). Figure 1 shows treatment allocated and actually received. Compliance was good, and treatment was prompt (appendix p 10). Among those allocated to CAS, 1578 (87%) had it within 1 year, at median 14 days (IQR 4–33) after randomisation, 101 (6%) crossed over to CEA, and 106 (6%) had no intervention. Among those allocated CEA, 1668 (92%) had it within a year, again at median 14 days (IQR 4–33) after randomisation, 48 (3%) crossed over to CAS, and 78 (4%) had no intervention. Reasons for crossing over from CAS to CEA included finding that the stenosis was highly calcified or that the carotid artery was more tortuous than anticipated. Reasons for crossing over from CEA to CAS included the patient's or doctor's preference, or reluctance to undergo general anaesthesia. Only about half the CAS procedures were done by a radiologist; most of the others were done by vascular surgeons. The techniques and drug treatment of those having CAS and CEA as their first carotid procedure after randomisation is described in the appendix (pp 11–14); CAS was usually accompanied by double antiplatelet therapy.

Table 2 describes the procedural hazards in those who had an intervention, subdivided both by the first intervention actually undertaken and by the random allocation; in both analyses, the findings were similar. Among those who actually had CAS or actually had CEA, there was a small excess of non-disabling strokes after CAS (45 *vs* 32, including 15 *vs* six with no residual symptoms at all [mRS score 0]) and a small excess of myocardial infarction after CEA (four *vs* 13), but the overall risk of death or disabling stroke was similar: CAS 1·0% (17 of 1653) versus CEA 0·9% (15 of 1788). The risk of stroke within 30 days was similar between CAS done by radiologists and other operators (appendix p 15). Nine procedural strokes were haemorrhagic (six CAS *vs* three CEA, including one *vs* one disabling). For patients without complications, mean hospital stay was 1 day shorter after CAS than after CEA (4·2 days, SD 9·0, *vs* 5·4 days, SD 10·1); about two thirds of the procedural events occurred before these medians (appendix p 16). Of 1788 with CEA as the first intervention, 96 (5·4%) had cranial nerve palsy described on the 1-month form (33 n.XII, 29 n.VII, 23 n.X, six n.V, three n.XI, one n.VIII, and one n.IX). CAS did not cause cranial nerve palsy.

**Table 2:** Death, stroke, or MI within 30 days of first carotid procedure*

		**Allocated CAS (n=1811)**	**Allocated CEA (n=1814)**	**p value**	**Had CAS first**	**Had CEA first**
Had no carotid procedure		106	78	..	..	..
Had a carotid procedure†		1705	1736	..	1653	1788
Worst procedural stroke, mRS score						
	6 (fatal)	7	5	0·77	6	6
	3–5 (disabling)	6	7	1·00	8	5
	2	9	9	1·00	9	9
	1	23	15	0·25	21	17
	0	16	5	0·03	15	6
	0–2 (non-disabling)	48 (2·7%)	29 (1·6%)	0·03	45 (2·7%)	32 (1·8%)
Subtotal: any stroke		61 (3·6%)	41 (2·4%)	0·06	59 (3·6%)	43 (2·4%)
MI						
	Fatal	0	4	0·13	0	4
	Non-fatal	5	8	0·58	4	9
Subtotal: any MI		5 (0·3%)	12 (0·7%)	0·15	4 (0·2%)	13 (0·7%)
Other death‡		2	2	1·00	3	1
Death, MI, or any stroke		67 (3·9%)	55 (3·2%)	0·26	65 (3·9%)	57 (3·2%)
Death or any stroke		63 (3·7%)	47 (2·7%)	0·12	62 (3·8%)	48 (2·7%)
Death or disabling stroke		15 (0·9%)	18 (1·0%)	0·77	17 (1·0%)	16 (0·9%)

Data are n or n (%), unless otherwise specified. CAS=carotid artery surgery. CEA=carotid endarterectomy. MI=myocardial infarction. mRS=modified Rankin Scale.

* First carotid procedure undergone after randomisation.

† Denominator for percentages.

‡ One groin haemorrhage after CAS, one unrelated trauma death after CAS, one cervical haemorrhage after CEA, and one generalised sepsis (allocated CEA but got CAS).

The mean duration of follow-up was 4·9 years, SD 3·1 (range 0–12; figure 1). Annual follow-up is still continuing, with wide use of antithrombotic, antihypertensive, and lipid-lowering therapy (appendix p 17) and no material differences in usage between those allocated CAS and CEA. Among those who had a carotid procedure without a stroke, slightly more of those allocated CAS than of those allocated CEA had a stroke during long-term follow-up (table 3).

**Table 3:** Non-procedural strokes during follow-up

		**Allocated CAS (n=1811)**	**Allocated CEA (n=1814)**
Procedural stroke or death		63	47
No procedural stroke or death*		1748	1767
Worst non-procedural stroke, by mRS score†			
	6 (fatal)	16 (0·9%)	20 (1·1%)
	3–5 (disabling)	28 (1·6%)	25 (1·4%)
	2	9	5
	1	23	17
	0	15	12
	0–2 (non-disabling)	47 (2·7%)	34 (1·9%)
Total: any non-procedural stroke		91 (5·2%)	79 (4·5%)

CAS=carotid artery surgery. CEA=carotid endarterectomy. mRS=modified Rankin Scale.

* Denominator for percentages; this includes patients with no procedure.

† Corresponding numbers of first non-procedural strokes (CAS *vs* CEA): 12 versus 17 fatal, 23 versus 22 disabling, 56 versus 40 non-disabling, and totals 91 versus 79; these totals include 15 strokes (seven CAS and eight CEA) with neither procedure beforehand, of which five were in the first month (ie, shortly after randomisation, while awaiting treatment) and ten were later (at mean 25 months after entry).

**Figure 2:**
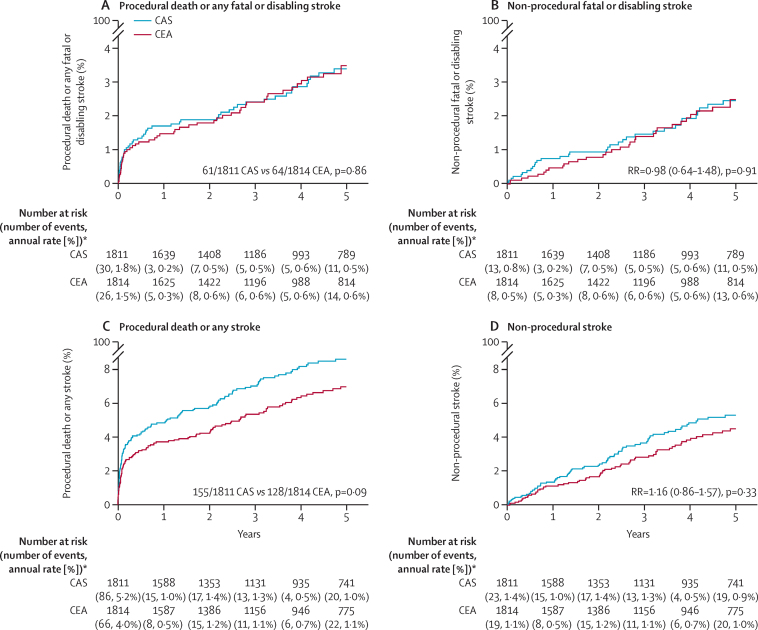
Kaplan-Meier estimates of 5-year outcomes among asymptomatic patients randomly allocated to CAS versus CEA CAS=carotid artery stenting. CEA=carotid endarterectomy. *Last rate is after year 5 (and all three procedural strokes due to a second carotid procedure were after year 5).

Figure 2 displays the main findings from ACST-2 on 5-year outcome, comparing all those allocated CAS versus all those allocated CEA, regardless of their actual treatment (intention-to-treat analyses). The left panels include procedural events, and the right panels show only non-procedural strokes. The upper two panels describe fatal or disabling events, and within them there was no difference between CAS and CEA in the incidence of fatal or disabling stroke. The lower two panels include all strokes, and within them the differences between CAS and CEA chiefly reflect differences in the incidence of non-disabling stroke. In figure 2C, the total numbers with an event were 155 of 1811 CAS versus 128 of 1814 CEA; this excludes the five versus eight non-fatal procedural myocardial infarcts (table 2). In figure 2D the non-procedural stroke incidence RR was 1·16 (95% CI 0·86–1·57, p=0·33), based on 91 versus 79 strokes (32 *vs* 21 definitely ipsilateral plus 59 *vs* 58 not; appendix p 18 provides similar analyses for these ipsilateral strokes).

**Figure 3:**
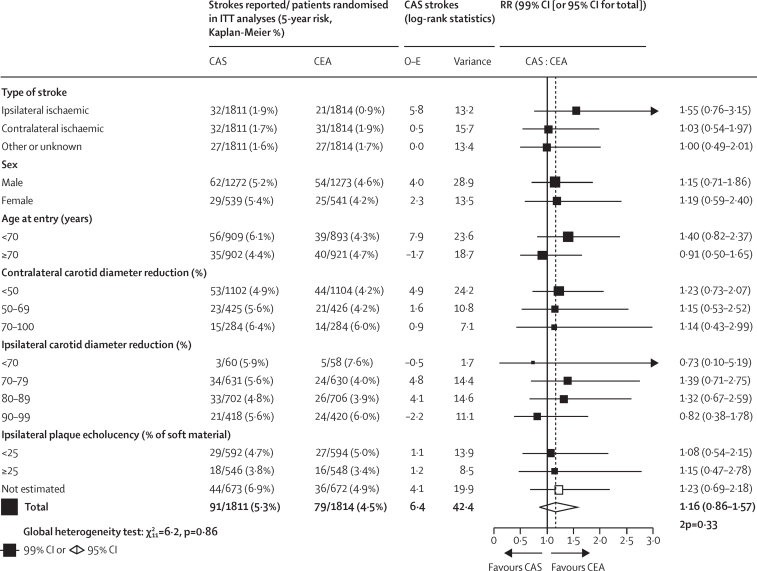
Subgroup analyses of long-term non-procedural stroke rates, by random allocation to CAS or to CEA 2p=two-sided p value. CAS=carotid artery stenting. CEA=carotid endarterectomy. ITT=intention-to-treat. O–E=log-rank observed minus expected. RR=rate ratio.

Subdivision of the overall findings for non-procedural stroke by various baseline characteristics found that these were of little prognostic relevance and yielded no significant evidence of heterogeneity of the treatment effect with respect to age, sex, stenosis, plaque echolucency, or any other factor (figure 3). Combining procedural and other deaths, the random allocation to CAS or CEA had no significant effect on overall stroke mortality (23 CAS *vs* 25 CEA stroke deaths; RR 0·93, 95% CI 0·53–1·63; p=0·80; Table 2, Table 3) or on all-cause mortality (330 *vs* 313 deaths, 92% of which were not from stroke; 1·04, 0·89–1·21; p=0·63).

## Discussion

This trial does not address the question of whether, or when, a carotid intervention would be appropriate, as it was restricted to patients in whom intervention was considered necessary, and all participants were to receive CAS or CEA. Previous trials of CEA versus no carotid procedure in asymptomatic patients had, however, already shown that CEA approximately halves the subsequent incidence of disabling or fatal stroke.^4^, ^5^ They had also shown that this approximate halving of non-procedural stroke rates by CEA did not depend significantly on age, sex, or use of effective medical treatment (which also reduces stroke rates substantially).

The main finding from the ACST-2 trial of CAS versus CEA is that the effects of the two procedures on disabling or fatal events are approximately equal in terms of procedural hazards (about 1% for each treatment, in line with findings from large, representative registries) and of 5-year disabling stroke rates (which were about 0·5% per year with either procedure, suggesting that they would have been about 1% per year with neither procedure).^4^, ^5^ Non-disabling procedural stroke rates appeared to be slightly higher with CAS, again consistent with recent results from registries. The chief limitation of ACST-2 is the study size; this is the largest carotid intervention trial yet conducted, but still it must be considered together with all other trials of CAS versus CEA.

Duplex Doppler identified and excluded from randomisation any patient whose stenosis was not thought to justify intervention. Patients then had CT or MRI to confirm that CAS and CEA were both practicable. The duplex Doppler stenosis assessment at entry was done by locally validated criteria but sufficed to show that the large majority had 70–99% stenosis, and that the measured degree of stenosis was of little relevance to prognosis or to the treatment comparison. Although the population studied is heterogeneous, and the trial did not monitor risk factor control during follow-up, this does not bias the randomised comparison between the two procedures.

Patients and doctors who take part in trials may well be atypical in various ways, so the absolute procedural hazards for typical patients treated by typical doctors may be better assessed by considering thoughtfully the evidence from large, recent, representative registries or routine health-care databases rather than by considering just the evidence from randomised trials. In the national German registry, asymptomatic patients undergoing CAS or CEA during 2014–19 had in both cases an in-hospital risk of disabling stroke or death of 0·7%, with median time to discharge of 4–5 days^3^ (appendix p 9). A risk of 0·7% within 4–5 days suggests a 30-day risk of disabling stroke or death of about 1% for each procedure, which is similar to that in ACST-2. Both in the German registry data and in ACST-2, CAS was associated with a slightly greater risk than CEA of non-disabling stroke.

Treatment was equally prompt in both study arms, so procedural endpoints could be defined as those within 30 days of the procedure (rather than within a fixed time since randomisation, as in some previous trials). The chief emphasis was on stroke, particularly disabling stroke, as procedural myocardial infarction was much less common than expected in the protocol, but there was no evidence that myocardial infarction had been underestimated. Cranial nerve damage following CEA was monitored only at 1 month, as it is usually either transient or not substantially disabling.^11^

Long-term follow-up sought only symptomatic strokes and did not image patients without symptoms. Follow-up thus far is to a mean of only 5 years, and properly informed medical decisions and reliable health economic evaluations could require even longer follow-up.

**Figure 4:**
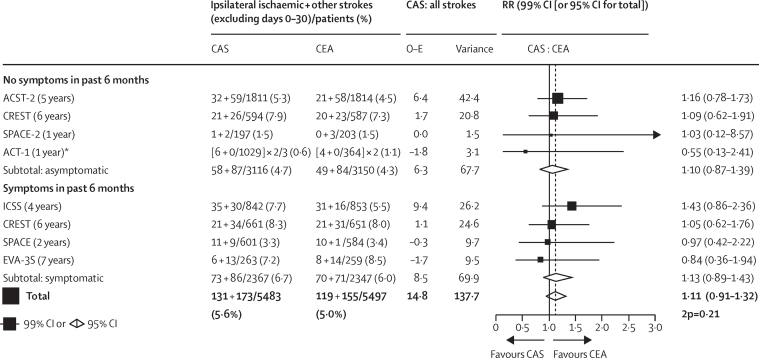
Trials of CAS versus CEA for asymptomatic or symptomatic carotid stenosis—ITT analyses of non-procedural strokes (ipsilateral ischaemic stroke plus other strokes) The figure excludes the 13 smaller trials (all evenly randomised) identified by the 2020 Cochrane review, which reported that they had, in total, 30 non-procedural strokes in 692 patients with CAS versus 24 non-procedural strokes in 715 patients with CEA. A repeat search on July 31, 2021, re-using the Cochrane search criteria identified no more trials of CAS versus CEA. 2p=two-sided p value. CAS=carotid artery stenting. CEA=carotid endarterectomy. ITT=intention-to-treat. O–E=log-rank observed minus expected. Var (O–E)=variance of (O–E). *ACT-1 allocated patients in a 3:1 ratio; for balance, therefore, it contributes two thirds of its CAS cases and double its CEA cases to the subtotal and the total case numbers; its main report provides exact numbers only for ischaemic strokes within 1 year.

If the disabling or fatal procedural hazards of CAS and CEA are similar, even moderate differences in long-term efficacy against stroke could be medically important. Analyses of registries or routine health-care databases cannot reliably assess moderate differences in long-term stroke rates, as there may well be systematic differences between the types of patients who undergo CAS and CEA that cannot be sufficiently controlled by mathematical modelling or propensity matching. Large-scale randomised evidence is necessary,^4^ and the present trial has more than doubled the number of asymptomatic patients in trials of CAS versus CEA.^12^, ^13^, ^14^ However, the randomised evidence from both asymptomatic and symptomatic patients is relevant.^14^, ^15^, ^16^, ^17^, ^18^ This is summarised in figure 4, the main aim of which is to see whether the results among the two types of patient can help reinforce each other, rather than to seek differences between them.

With ACST-2 included, there is now as much evidence among asymptomatic as among symptomatic patients, and the findings in both types of patient are remarkably similar, with CEA slightly but non-significantly better than CAS, at least for non-disabling stroke. Overall, the ratio (CAS *vs* CEA) of long-term stroke incidence rates is 1·11 (95% CI 0·91–1·32; p=0·21). As previous studies have shown successful CEA to be substantially protective,^4^, ^5^ this RR of 1·11 (which includes the ACST-2 result) shows that the protective effects of CAS and CEA are similar for at least the first few years. Further follow-up of ACST-2 and other trials will provide additional evidence on the durability of their protective effects.

## Data sharing

Follow-up of deaths and strokes will continue until 2026, when the final results will be reported and the dataset shared under Nuffield Department of Population Health (NDPH) data access policies.

## Declaration of interests

We declare no competing interests.
