# Supplementary material for: Second asymptomatic carotid surgery trial (ACST-2): a randomised comparison of carotid artery stenting versus carotid endarterectomy
Source: Lancet. 2021 Sep 18;398(10305):1065–73. doi: 10.1016/S0140-6736(21)01910-3 (PMC8473558; doi:10.1016/S0140-6736(21)01910-3)
Supplement: Supplementary appendix [file mmc1.pdf]

THE LANCET

Supplementary appendix

This appendix formed part of the original submission and has been peer reviewed. We post it as supplied by the authors.

Supplement to: Halliday A, Bulbulia R, Bonati LH, et al. Second asymptomatic carotid surgery trial (ACST-2): a randomised comparison of carotid artery stenting versus carotid endarterectomy. *Lancet* 2021; published online Aug 29. http://dx.doi.org/10.1016/S0140-6736(21)01910-3.

Online material for

Second asymptomatic carotid surgery trial (ACST-2): randomised comparison of carotid artery stenting vs carotid endarterectomy

Contents list (click on an item to jump to it)

Page

3 ACST-2 principal investigators, committees, staff, support, and collaborators

9 *Webtable W1:* In-hospital risk of stroke or death following CAS or CEA during 2014-19 in Germany, from the IQTiG mandatory nationwide registry of carotid procedures

10 *Webfigure W1:* 5-year allocated and actual use of CAS or CEA

11 *Webtable W2:* Carotid stent used in 1588 patients with CAS as first carotid procedure after randomisation

12 *Webtable W3:* Cerebral protection in 1588 patients with CAS as first carotid procedure after randomisation

13 *Webtable W4:* Anaesthetic type, patching and shunting in 1731 patients with CEA as first carotid procedure after randomisation

14 *Webtable W5:* Drug therapy 1 month after CAS or CEA

15 *Webtable W6:* Hazards of CAS as first carotid procedure after randomisation, according to the specialty of the operator

16 *Webfigure W2:* Death or stroke with onset ≤30 days from the first carotid intervention, by procedure actually performed (A) Disabling stroke or death; (B) Any stroke or death

17 *Webfigure W3:* Drug therapy during follow-up, by year since successful CAS or CEA

18 *Webfigure W4:* Ipsilateral non-procedural strokes, either including or excluding procedural events (A & B) Disabling or fatal events; (C & D) Events of any severity

19 ACST-2 protocol (original version)

Principal Investigators
Alison Halliday, Richard Bulbulia, Leo Bonati, Richard Peto, Hongchao Pan

Steering committee
J Potter (chair), L Bonati, R Bulbulia, C Davies (to 2017), HH Eckstein, B Farrell, M Flather, W Gray, A Halliday, S MacDonald (to 2017), A Mansfield, B Mihaylova, H Pan, R Peto, K Rahimi, D Simpson, D Thomas

Data monitoring committee
P Sandercock (chair), R Gray, A Molyneux, CP Shearman

Endpoint review committee
P Rothwell (chair), A Belli, W Herrington, P Judge, P Leopold, M Mafham, K Rahimi

Technical Management committee
M Gough (chair), P Cao, S MacDonald (to 2017)

Writing committee
A Halliday, R Bulbulia, L Bonati, R Peto, H Pan

ACST-2 staff and support
Clinical Trial Service Unit, Nuffield Department of Population Health, Oxford, UK — V Bari, C Berry, S Bradshaw, W Brudlo, A Clarke, J Chester, R Cox, A Cradduck-Bamford, S Fathers, K Gaba, M Gray, E Hayter, C Holliday, M Lay, S le Conte, R Kurien, Z Madgwick, D Morris, A Munday, S Pickworth, W Ostasz, M Poorthuis, S Richards, L Teixeira, S Tochlin, L Tully, C Wallis, M Willett, A Young

Collaborating clinicians
Italy (867 patients): Istituto Auxologico Italiano IRCCS, Milan (R Casana, C Malloggi, A Odero Jr, V Silani, G Parati); Santa Maria Hospital, Reggio Emilia (G Malchiodi, G Malferrari, F Strozzi, N Tusini, E Vecchiati); St. Agostino-Estense Hospital, Modena (G Coppi, A Lauricella, R Moratto, R Silingardi, J Veronesi, A Zini); Mauriziano Hospital, Turin (E Ferrero, M Ferri, A Gaggiano, C. Labate, F Nessi, D Psacharopulous, A Viazzo); Policlinico San Donato, Milan (G Malacrida, D Mazzaccaro, G Meola, A Modafferi, G Nano, MT Occiuto, P Righini, S Stegher); University Hospital of Trieste, Trieste (S Chiarandini, F Griselli, S Lepidi, F Pozzi Mucelli, M Naccarato, M D'Oria, B Ziani); University Hospital of Bologna, Bologna (A Stella, M Dieng, G Faggioli, M Gargiulo, S Palermo, R Pini, GM Puddu, A Vacirca); University Hospital of Bari, Bari (D Angiletta, D Marinazzo, G Mastrangelo, G Regina, R Pulli); Policlinico di Monza, Monza (P Bianchi, L Cireni, E Coppi, R Pizzirusso, F Scalise, G Sorropago, V Tolva); University Hospital of Perugia, Perugia (P Cao, V Caso, E Cieri, P DeRango, L Farchioni, G Isernia, M Lenti, G Parlani, G Pupo, G Pula, G Simonte, F Verzini); University of Insubria, Varese (F Carimati, ML Delodovici, F Fontana, G Piffaretti, M Tozzi); IRCCS Istituto Clinico Humanitas, Milan (E Civilini, G Poletto, B Reimers); ACO San Filippo Neri, Rome (B Praquin, S Ronchey); University La Sapienza, Rome (L Capoccia, W Mansour, E Sbarigia, F Speziale, P Sirignano, D Toni); St Anna University Hospital, Ferrara (R Galeotti, V Gasbarro, F Mascoli, T Rocca, E Tsolaki); Vittorio Emanuele Hospital, Catania (G Bernardini, E DeMarco, A Giaquinta, F Patti, M Veroux, P Veroux, C Virgilio); San Camillo Forlanini, Rome (N Mangialardi,

M Orrico); Policlinico Universitario Campus Bio-medico, Rome (V Di Lazzaro, N Montelione, F Spinelli, F Stilo); San Giacomo Hospital, Castelfranco Veneto (C Cernetti, S Irsara, G Maccarrone, D Tonello, A Visonà, B Zalunardo); San Giovanni Di Dio Hospital, Florence (E Chisci, S Michelagnoli, N Troisi); Mirano Hospital, Mirano Veneto (M Masato, M Dei Negri, A Pacchioni, S Saccà); SG Moscati Hospital, Avellino (G Amatucci, A Cannizzaro); San Paolo Hospital, Civitavecchia (F Accrocca, C Ambrogi, R Barbazza, G Marcucci, A Siani); Niguarda Hospital, Palermo (G Bajardi, G Savettieri); IRCCS Policlinico San Matteo, Pavia (A Argentieri, R Corbetta, A Odero, P Quaretti, Z Thyrion); Policlinico Santa Maria alla Scotte, Siena (A Cappelli, D Benevento, G De Donato, MA Mele, G Palasciano, D Pieragalli, A Rossi, C Setacci, F Setacci); IRCCS San Martino, Genoa (D Palombo, M C Perfumo); IRCCS Ca' Granda, Milan (E Martelli, A Paolucci, S Trimarchi); Cefalù Fondazione Istituto G Giglio (A Giaquinta, L Grimaldi, G La Rosa, D Mirabella, M Scialabba, L Sichel, P Veroux); San Francesco Hospital, Sardegna (CL Di Angelo, GF Fadda, H Kasemi, M Marino); IRCCS Agostino Gemelli, Rome (F Burzotta, FA Codispoti, AM Ferrante, G Tinelli, Y Tshomba, Vincenzoni).

United Kingdom (470 patients): Freeman Hospital, Newcastle (D Amis, D Anderson, M Catterson, M Clarke, M Davis, A Dixit, A Dyker, G Ford, R Jackson, S Kappadath, D Lambert, T Lees, S Louw, S Macdonald, J. McCaslin, N Parr, R Robson, G Stansby, L Wales, V Wealleans, L Wilson, M Wyatt); Kent and Canterbury Hospital, Kent (H Baht, I Balogun, I Burger, T Cosier, L Cowie, G Gunathilagan, D Hargroves, R Insall, S Jones, H Rudenko, N Schumacher, J Senaratne, G Thomas, A Thomson, T Webb); Bishop Auckland Hospital, Durham (E Brown, B Esisi, A Mehrzad); Nottingham University Hospital, Nottingham (S MacSweeney, N McConachie, A Southam, W Sunman); Hull Royal Infirmary, Hull (A Abdul-Hamiq, J Bryce, I Chetter, D Ettles, R Lakshminarayan, K Mitchelsonm, C Rhymes, G Robinson, P Scott, A Vickers); Manchester University Hospitals Foundation Trust (J Akyea-Mensah, R Ashleigh, S Ball, S Butterfield, A Chrisopoulou, E Gamble, J Ghosh, E Keens, CN McCollum, A Phair, S Rogers, JV Smyth, M Welch, S Welsh, L Wolowczyk); The Royal Preston Hospital, Preston (M Donnelly, S D'Souza, A Egun, B Gregory, C Kelly, S Punekar, AM Rahi, S Raj, D Seriki, G Thomson, J Thomas); Southend University Hospital, Southend (J Brown, R Durairajan, I Grunwald, P Guyler, P Harman, M Jakeways, C Khuoge, A Kundu, T Loganathan, N Menon, RO Prabakaran, D Sinha, V Thompson, S Tysoe, S Tysoe); John Radcliffe Hospital, Oxford (D Briley, C Darby, A Halliday, A Handa, L Hands, D Howard, W Kuker, J Perkins, U Schulz, R Teal); Sunderland Royal Hospital, Sunderland (D Barer, A Brown, S Crawford, P Dunlop, R Krishnamurthy, N Majmudar, D Mitchell, M Myint, R O'Brien, J O'Connell, N Sattar, S Vetrivel); Sheffield Vascular Institute, Sheffield (J Beard, T Cleveland, P Gaines, J Humphreys, A Jenkins, C King, D Kusuma, R. Lindert, R Lonsdale, R Nair, S Nawaz, F Okhuoya, D Turner, G Venables); Royal Victoria Infirmary, Newcastle upon Tyne (P Dorman, A Hughes, D Jones, D Mendelow, H Rodgers, A Raudoniitis); Walton Centre, Liverpool (P Enevoldson, H Nahser, I O'Brian, F Torella, D Watling, R White); Cheltenham General Hospital, Cheltenham (P Brown, R Bulbulia, D Dutta, L Emerson, P Hillout, S Kulkarni, J Morrison, K Poskitt, F Slim, S Smith, A Tyler, J Waldron, M Whyman); Great Western Hospital NHS Foundation Trust, Wiltshire (M Bajoriene, L Baker, A Colston, B Eliot-Jones, G Gramizadeh, S Kulkarni, C Lewis-Clarke, L McCafferty, D Oliver, D Palmer, A Patil, S Pegler, G Ramadurai,

A Roberts, T Sargent, S Siddegowda, R Sigh-Ranger, A Williams, L Williams, S Windebank, T Zuromskis); Luton and Dunstable University Hospital (L Alwis, J Angus, A Asokanathan, C Fornolles, D Hardy, S Hunte, F Justin, D Phiri, M Mitaboauana-Kibou, L Sekaran, S Sethuraman, M Tate); St Mary's Hospital, London (C Bicknell, J Chataway, N Cheshire, C Eley, R Gibbs, M Hamady, B Hazel, A James, M Jenkins, N Khanom, A Lacey, M Mireskandari, J O'Reilly, A Pereira, T Sachs, J Wolfe); University Hospital of Durham (E. Brown, P Davey, A. Mehrzad, G. Rogers, G Smith, G Tervit); James Cook Hospital, Middlesbrough (I Nichol, A Parry, G Young); Derriford Hospital, Plymouth (S Ashley, J Barwell, F Dix, A Mohd Nor, C Parry); North Cumbria University Hospital, Carlisle (A Birt, P Davies, J George, A Graham, L Jonker, T Joseph, N Kelsall, C Potts, T Wilson); The Royal London Hospital, London (J Crinnion, L Cuenoud).

Serbia (326 patients): Cardiovascular Centar Dedinje, Belgrade (N Aleksic, S Babic, N Ilijevski, D Radak, D Sagic, S Tanaskovic); Serbian Clinical Centre, Belgrade (M Colic, V Cvetic, L Davidovic, D Jovanovic, I Koncar, P Mutavdzic, M Sladojevic, I Tomic).

Germany (253 patients): University Medical Center Hamburg-Eppendorf (ES Debus, U Grzyska, D Otto, G Thomalla); University of Dresden, Dresden (J Barlinn, J Gerber, K Haase, C Hartmann, S Ludwig, V Pütz, C Reeps, C Schmidt, N Weiss, S Werth, S Winzer); University Hospital of Jena, Jena (J Gemper, A Guenther, B Heiling, E Jochmann, P Karvouniari, C Klingner, T Mayer, J Schubert, F Schulze-Hartung, J Zanow); Universitätsklinikum Leipzig/Park Hospital, Leipzig (Y Bausback, F Borger, S Botsios, D Branzan, S Braeunlich, H Holzer, J Lenzer, C Piorkowski, N Richter, J Schuster, D Scheinert, A Schmidt, H Staab, M Ulrich, M Werner); Department for Vascular and Endovascular Surgery, University Hospital, Technical University of Munich (TUM), Munich (H Berger, G Biro, HH Eckstein, M Kallmayer, K Kreiser, A Zimmermann); St Franciskus Hospital, Munster (B Berekoven, K Frerker, V Gordon, G Torsello); Städtisches Klinikum Karlsruhe (S Arnold, C Dienel, M Storck); Singen General Hospital, Singen (B Biermaier, HM Gissler, C Klotzsch, T Pfeiffer, R Schneider, L Soehl, M Wennrih); Universitätsmedizin Mannheim (A Alonso, M Keese, C Groden); Pius Hospital, Oldenburg (A Coster, A Engelhardt, CM Ratusinski).

Sweden (244 patients): Sodersjukhuset Hospital, Stockholm (B Bengt, M Delle, J Formgren, P Gillgren, L Jarl, TB Kall, P Konrad, J Malmstedt, N Nyman, C Skioldebrand, J Steuer, R Takolander); Skåne University Hospital, Malmö (S Acosta, K Bjorses, K Brandt, N Dias, A Gottsater, J Holst, T Kristmundsson, T Kuhme, T Kolbel, B Lindblad, M Lindh, M Malina, T Ohrlander, T Resch, V Rönnle, B Sonesson, M Warvsten, Z Zdanowski); Helsingborg Hospital, Helsingborg (E Campbell, P Kjellin, H Lindgren, J Nyberg, B Petersen, G Plate, H Parsson, P Qvarfordt).

Russia (187 patients): E. Meshalkin National Medical Research Center, Novosibirsk (P Ignatenko, A Karpenko, V Starodubtsev); VA Almazov National Medical Research Centre, St Petersburg (M Chernyavsky, M Golovkova, B Komakha, N Zherdev); I. I. Dzhanelidze Research Institute, St Petersburg (A Belyasnik, P Chechulov, D Kandyba, I Stepanischev).

Hungary (163 patients): Semmelweis University, Department of Vascular and Endovascular Surgery, Budapest (C Csobay-Novak, E Dósa, L Entz, B Nemes, Z Szeberin); Albert Szent-Györgyi Medical Centre, Szeged (P Barzo, M Bodosi, E Fako, B Fulop, T Nemeth, S Pazdernyik, K Skoba, E Voros).

Greece (117 patients): Department of Vascular Surgery, University Hospital of Larissa (E Chatzinikou, A Giannoukas, C Karathanos, S Koutsias, G Kouvelos, M Matsagkas, S Ralli, C. Rountas, N Rousas, K Spanos); Attikon University Hospital, Athens (E Brountzos, Y Kakisis, A Lazaris, KG Moulakakis, L Stefanis, G Tsivgoulis, S Vasdekis); Evangelismos General Hospital, Athens (C Antonopoulos, I Bellenis, D Maras, A Polydorou, V Polydorou, A Tavernarakis); IASO, Athens (N Ioannou, M Terzoudi);Democritus University of Thrace, Thrace (M Lazarides, M Mantatzis, K Vadikolias).

Poland (116 patients): University of Medical Sciences, Poznań (L Dzieciuchowicz, M Gabriel, Z Krasinski, G Oszkinis, F Pukacki, M Slowinski, MG Stanisic, R Staniszewski, J Tomczak, M Zielinski); Mazovian Brodnowski Hospital, Warsaw (P Myrcha, D Różański); Regional Specialist Hospital, Wroclaw (S Drelichowski, W Iwanowski, K Koncewicz); Medical University Hospital of Warsaw (P Bialek, Z Biejat, W Czepel, A Czlonkowska, A Dowzenko, J Jedzrejewska, A Kobayashi, J Leszezyuski, A Malek, J Polanski, R Proczka, M Skorski, M Szostek); Central Hospital of Internal Affairs and Administration Ministry, Warsaw (P Andziak, M Dratwicki, R Gil, M Nowicki, J Pniewski, J Rzezak, P Seweryniak); Bielanski Hospital, Warsaw (P Dabek, M Juszynski, G Madycki, B Pacewski, W Raciborski, P Slowinski, W Staskziewicz).

Czechia (109 patients): Ceske Budejovice City Hospital (M Bombic, V Chlouba, J Fiedler, K Hes, P Košťál, J Sova); 2nd Department of Surgery, St. Anne's University Hospital, Brno and Faculty of Medicine, Masaryk University, Brno (Z Kriz, M Privara, M Reif, R Staffa, R Vlachovsky, B Vojtisek); Ostrava Poruba City Hospital (T Hrbac, M Kuliha, V Prochazka, M Roubec, D Skoloudik); Central Military Hospital, Prague (D Netuka, A. Steklacova); Regional Hospital, Liberec (V Beneš 3rd, P Buchvald, L Endrych, M Šercl).

Brazil (107 patients): Hospital das Clínicas da Faculdade de Medicina da USP (W Campos Jr, I Benaduce Casella, N de Luccia, A Echaime Vallentsits Estenssoro, B Neves, C Presti, P Puech-Leão, C Ricardo, ES da Silva, CJ Sitrângulo Jr, JA Tavares Monteiro, G Tinone); USP Medical School Hospital, Ribeirao Preto (M Bellini Dalio, EE Joviliano, OM Pontes Neto, M Serra Ribeiro).

Belgium (96 patients): University Hospital, Antwerp (P Cras, JMH Hendriks, M Hoppenbrouwers, P Lauwers, C Loos, L Yperzeele); University Hospital, Ghent (M Geenens, D Hemelsoet, I van Herzeele, F Vermassen); University Clinic St-Luc, Brussels (P Astarci, F Hammer, V Lacroix, A Peeters, R Verhelst); Centre Hospitalier De Mouscron (S Cirelli, P Dormal, A Grimonprez, B Lambrecht, P Lerut, E Thues); CHR Citadelle, Liège (G De Koster, Q Desiron, AM de Noordhout, D Malmendier, M Massoz, G Saad); Academic Hospital St Blasius, Dendermonde (M Bosiers, J Callaert, K Deloose).

Spain (91 patients): Guadalajara Hospital, Guadalajara (ME Blanco Cañibano, B Garcia Fresnillo, M Guerra Requena, PC Morata Barrado, M Muela Mendez, A Yusta Izquierdo); La Fe University and Polytechnic Hospital, Valencia (F Aparici-Robles, P Blanes, L García-Dominguez, R Matínez-López, M Miralles, J Tembel); Hospital Clínic, Barcelona (A Chamorro, J Macho, V Obach, V Riambau, L Sanroman).

Switzerland (79 patients): Basel University Hospital, Basel (FJ Ahlhelm, K Blackham, L Bonati, S Engelter, T Eugster, H Gensicke, L Guerke, P Lyrer, L Mariani, M Maurer, E Mujagic, M Müller, M Psychogios, P Stierli, C Stippich, C Traenka, T Wolff, B. Wagner, M Wieger); Cantonal Hospital Aarau, Aarau (S Clarke, M Diepers, E Groechenig, L Gürke, P Gruber, A Isaak, T Kahles, R Marti, K Nedeltchev, L Remonda, P Stierli, N Tissira, M Valenca Falcao).

Netherlands (76 patients): Utrecht University Medical Centre, Utrecht (GJ De Borst, AG Den Hartog, A Huibers, TH Lo, F Moll, R Toorop, HB Van Der Worp, EJ Vonken, LJ Kappelle); Leeuwarden Medical Center, Leeuwarden (O Jahrome, A Vos, W Schuiling); Haga Hospital, The Hague (H van Overhagen, RWM Keunen, B Knippenberg, JJ Wever); Rijnstate Hospital, Arnhem (JW Lardenoije, M Reijnen, L Smeets, S van Sterkenburg).

Austria (60 patients): Medical University of Innsbruck, Innsbruck (G Fraedrich, E Gizewski, I Gruber, M Knoflach, S Kiechl, B Rantner).

France (47 patients): St Joseph Hospital, Marseille (T Abdulamit, P Bergeron, Padovani, JC Trastour); Franciscan Hospital, Nimes (JM Cardon, A Le Gallou-Wittenberg); Henri-Mondor Hospital, Paris (E Allaire, JP Becquemin, F Cochennec, P Desgranges, H Hosseini, H Kobeiter, J Marzelle).

Canada (40 patients): Foothills Medical Centre, Calgary (MA Almekhlafi, S Bal, PA Barber, SB Coutts, AM Demchuk, M Eesa, M Gillies, M Goyal, MD Hill, ME Hudon, A Jambula, C Kenney, G Klein, M McClelland, A Mitha, BK Menon, WF Morrish, S Peters, KJ Ryckborst, G Samis, S Save, EE Smith, P Stys, S Subramaniam, G Sutherland, T Watson, JH Wong, L Zimmel).

Slovenia (37 patients): Maribor University Medical Centre, Maribor (V Flis, J Matela, K Miksic, F Milotic, B Mrdja, B Stirn, E Tetickovic); Izola General Hospital, Izola (M Gasparini, A Grad, I Kompara, Z Milosevic).

Estonia (36 patients): East Tallinn Central Hospital (V Palmiste, T Toomsoo).

Kazakhstan (21 patients): Sema Almaty Hospital, Almaty (B Aidashova, N Kospanov, R Lyssenko, D Mussagaliev).

Israel (17 patients): Rambam Medical Centre, Haifa (R Beyar, A Hoffman, T Karram, A Kerner, E Nikolsky, S Nitecki).

Bulgaria (16 patients): St Marina University Hospital, Varna (S Andonova, C Bachvarov, V Petrov).

Croatia (16 patients): Merkur University Hospital, Zagreb (I Cvjetko, V Vidjak); University Hospital Centre Zagreb (D Haluzan, M Petrunic).

China (12 patients): Peking Union Medical College, Beijing (Bao Liu, Chang-Wei Liu).

Slovakia (9 patients): Central Military Hospital, Ružomberok (D Bartko, P Beno, F Rusnak, K Zelenak).

Japan (9 patients): Sendai Medical Centre, Sendai (M Ezura, T Inoue, N Kimura, R Kondo, Y Matsumoto, H Shimizu); Kohnan Hospital, Sendai (H Endo, E Furui).

Norway (8 patients): Oslo University Hospital Rikshospitalet, Oslo (S Bakke, K Krohg-Sorensen, T. Nome, M Skjelland, B. Tennøe).

Portugal (3 patients): Hospital de Santa Marta, Lisbon (J Albuquerque e Castro, G Alves, F Bastos Gonçalves, A De Morais, AC Garcia, H Valentim, L Vasconcelos).

Argentina (2 patients): Buenos Aires Cardiovascular Institute, Buenos Aires (F Belcastro, F Cura, P Zaefferer).

Egypt (2 patients): Kasr Alainy School of Medicine, Cairo (F Abd-Allah, MH Eldessoki, H Heshmat Kassem, H Soliman Gharieb).

United States of America (1 patient): University of Toledo Medical Center, Toledo (V Kazan, M Nazzal, V Ramsey-Williams).

Ireland (1 patient): St James' Hospital, Dublin (M Colgan, SN Haider, J Harbison, P Madhavan, D Moore, G. Shanik).

Webtable W1: In-hospital risk of stroke or death following CAS or CEA during 2014-19 in Germany, from the IQTiG mandatory nationwide registry of carotid procedures and their outcomes

Notes:
- 97% of patients were classified as American Society of Anaesthesiology grades I, II, or III

- Median time to discharge of patients without adverse outcomes was 4-5 days

- Earlier IQTiG reports had found no material effect of age or sex on in-hospital stroke risk

	Asymptomatic patients having CAS	Asymptomatic patients having CEA	Symptomatic patients having CAS	Symptomatic patients having CEA
**Number of patients**	18,369	86,318	11,565	54,437
**Number with stroke or death before discharge:**				
- Disabling stroke or death	124 (0.7%)	587 (0.7%)	238 (2.1%)	881 (1.6%)
- Any stroke or death	330 (1.8%)	1226 (1.4%)	422 (3.6%)	1531 (2.8%)

Source: IQTiG https://iqtig.org/qs-verfahren/qs-karotis/ (accessed July 22, 2021)

Webfigure W1: **5-year allocated and actual use of CAS or CEA**

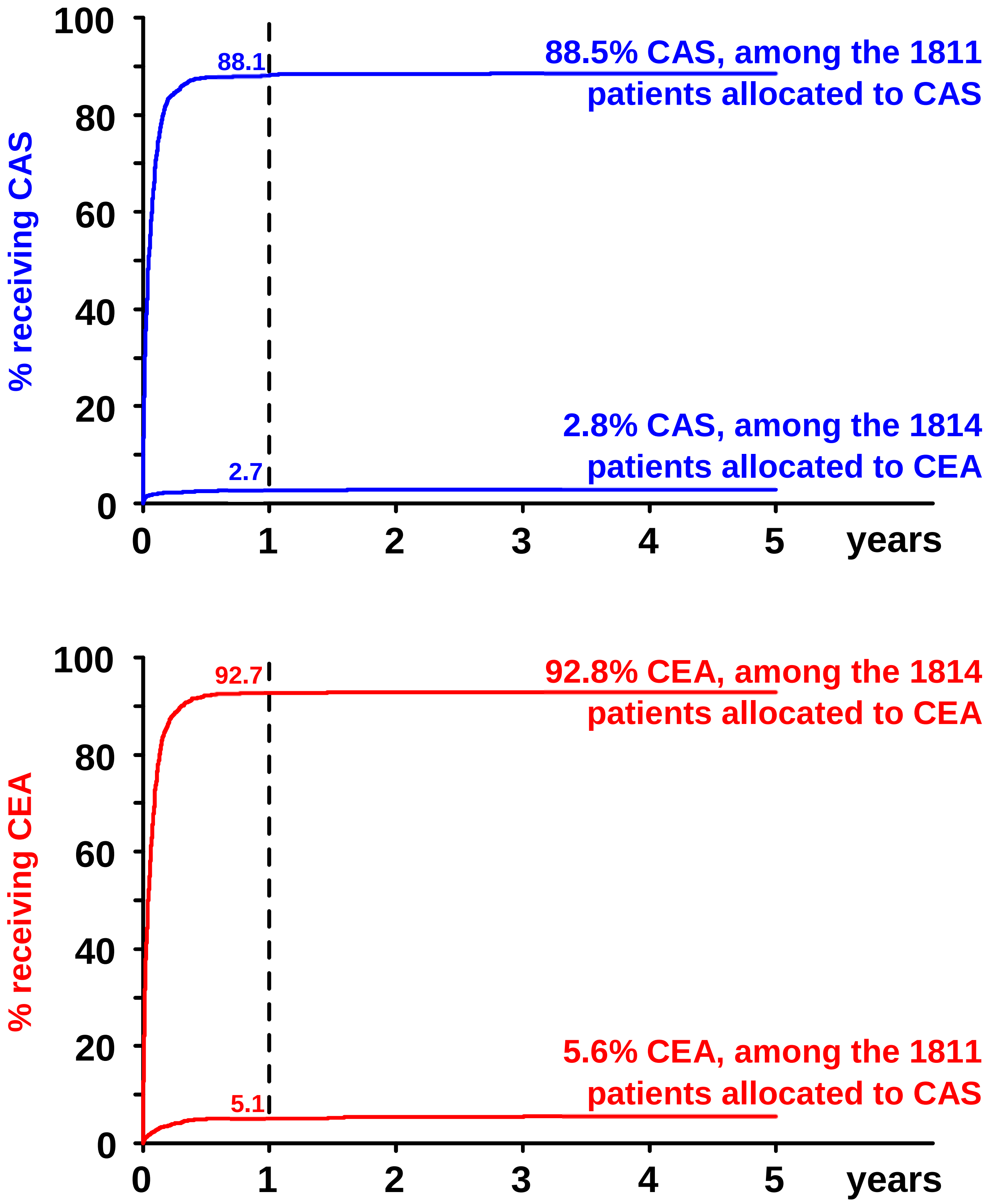

Webtable W2: Carotid stent used in 1588 patients with CAS as first carotid procedure after randomisation

	Stent name	Number	
**Closed cell**		**739**	**47%**
	Wallstent	469	
	XAct	261	
	Other	9	
**Open cell**		**482**	**30%**
	Precise	198	
	Protege Rx	144	
	Rx Acculink	122	
	Other	18	
**Hybrid**		**180**	**11%**
	Cristallo Ideale	170	
	Other	10	
**Membrane**		**176**	**11%**
	Roadsaver	109	
	CGuard	67	
**Other**		**11**	**1%**
**Total**		**1588**	**100%**

Webtable W3: **Cerebral protection in 1588 patients with CAS as first carotid procedure after randomisation**

	Name	Number	
**Filter**		**1097**	**69%**
	Filterwire	391	
	Emboshield	327	
	Spider	241	
	Accunet	78	
	Angioguard	55	
	Other	5	
**Proximal occlusion**		**238**	**15%**
	Moma	204	
	Gore flow reversal	27	
	Other	7	
**Distal balloon**		**9**	**1%**
**None***		**244**	**15%**
**Total**		**1588**	**100%**

* 8 centres decided generally not to use any cerebral protection device

Webtable W4: **Anaesthetic type, patching and shunting in 1731 patients with CEA as first carotid procedure after randomisation**

	**Patch used?**		**Shunt used?**	
	Yes	No	Yes	No
**Anaesthetic type**				
General	533	499	289	743
Local	203	484	47	643
Unknown	2	7	0	9
**Total**	738	993	336	1395

Webtable W5: Drug therapy 1 month after CAS or CEA

	**CAS done (n=1588)**	**CEA done (n=1731)**	**Total (n=3319)***
**Anti-hypertensive**	1359 (86%)	1505 (87%)	2864 (86%)
**Lipid-lowering**	1400 (88%)	1561 (90%)	2961 (89%)
**Year 2008-14**	617 (86%)	660 (88%)	1277 (87%)
**Year 2015-21**	783 (90%)	901 (92%)	1684 (91%)
**Anti-coagulant**	98 (6%)	124 (7%)	222 (7%)
**Anti-platelet**	1588 (100%)	1705 (99%)	3293 (99%)
**Aspirin and/or clopidogrel**	1582 (100%)	1680 (97%)	3262 (98%)
**Both aspirin and clopidogrel †**	1474 (93%)	367 (21%)	1841 (55%)

† As expected from treatment guidelines, there was much greater use of double anti-platelet therapy 1 month after stenting than 1 month after surgery, but this rarely persisted long-term.

Webtable W6: Hazards of CAS as first carotid procedure after randomisation, according to the specialty of the operator

	Death, any stroke, or MI within 30 days of CAS
**Radiologist**	28 / 830 (3.4%)
**Surgeon**	34 / 728 (4.7%)
**Other**	3 / 95 (3.2%)
**Total**	65 /1653 (3.9%)

Webfigure W2: **Death or stroke with onset within 30 days of the first carotid intervention, by procedure actually performed**
(A) Disabling or fatal events; (B) Any stroke or death

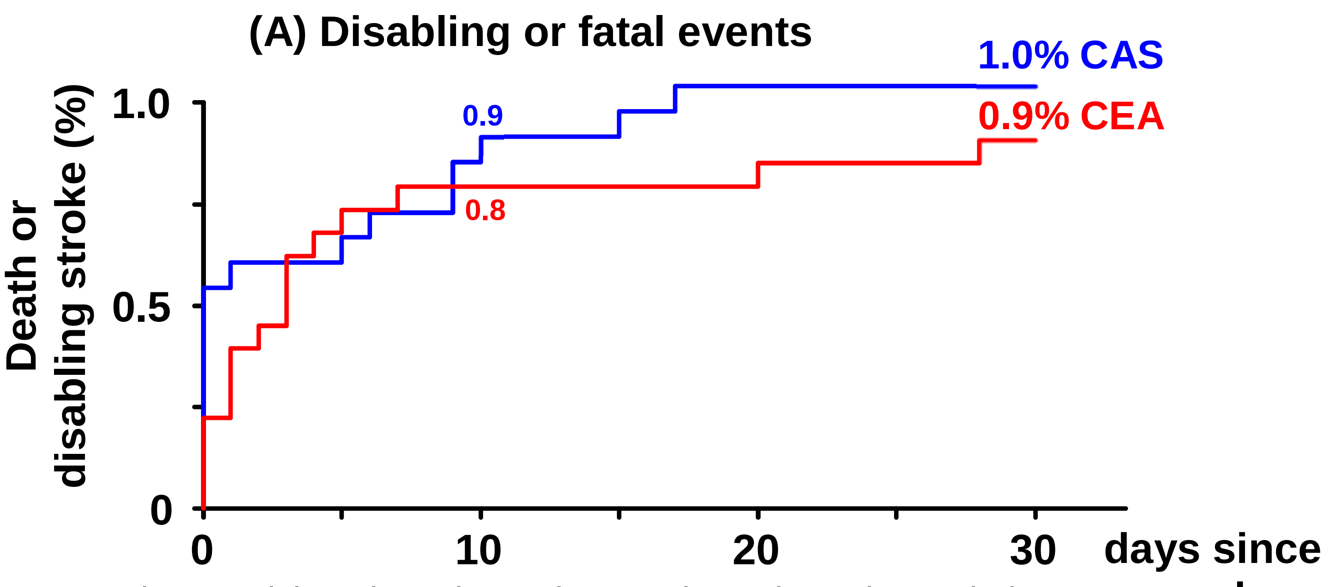

Numbers at risk and numbers of events in each 10-day period

CAS	1653	14	3	0
CEA	1788	14	0	2

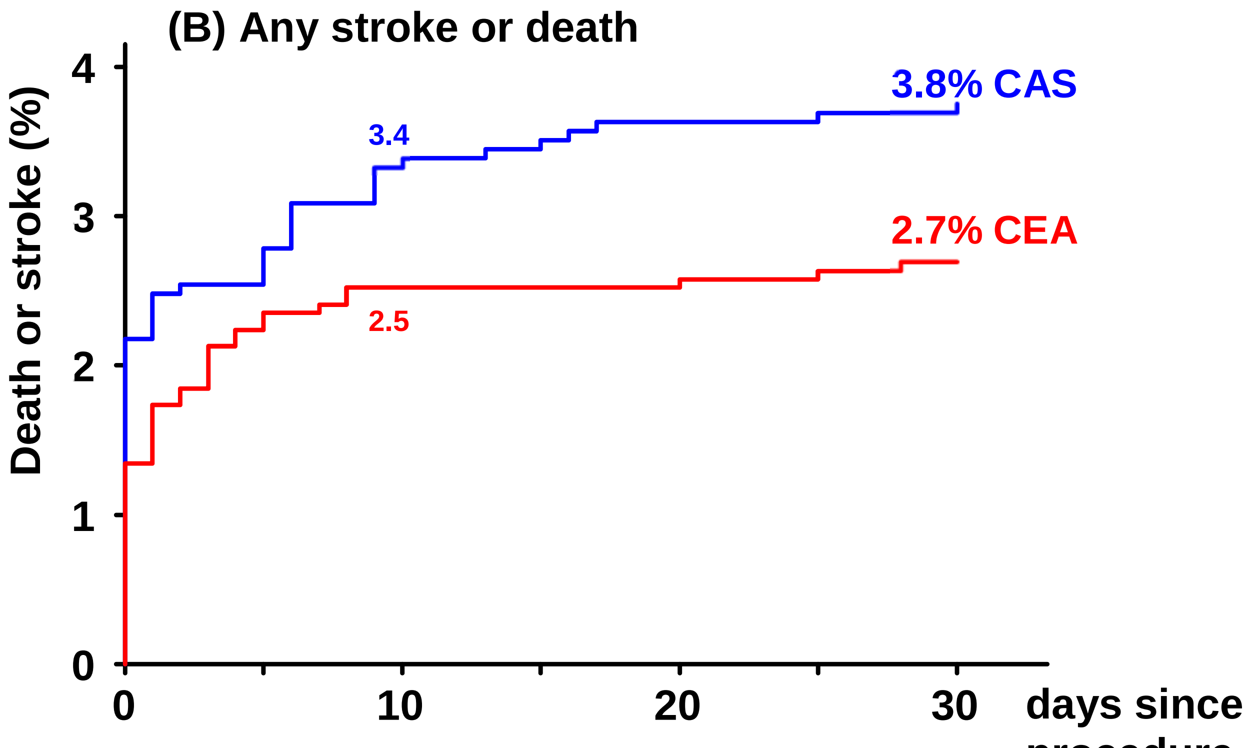

Numbers at risk and numbers of events in each 10-day period

CAS	1653	55	5	2
CEA	1788	45	0	3

***Webfigure W3:* Drug therapy during follow-up, by year since successful CAS or CEA**

% current usage reported at 1 month (year 0) and at years 1, 2, 3, 4, and 5 after study entry

Top: Anti-platelet (left) and anti-coagulant; *Bottom:* anti-hypertensive (left) and lipid-lowering

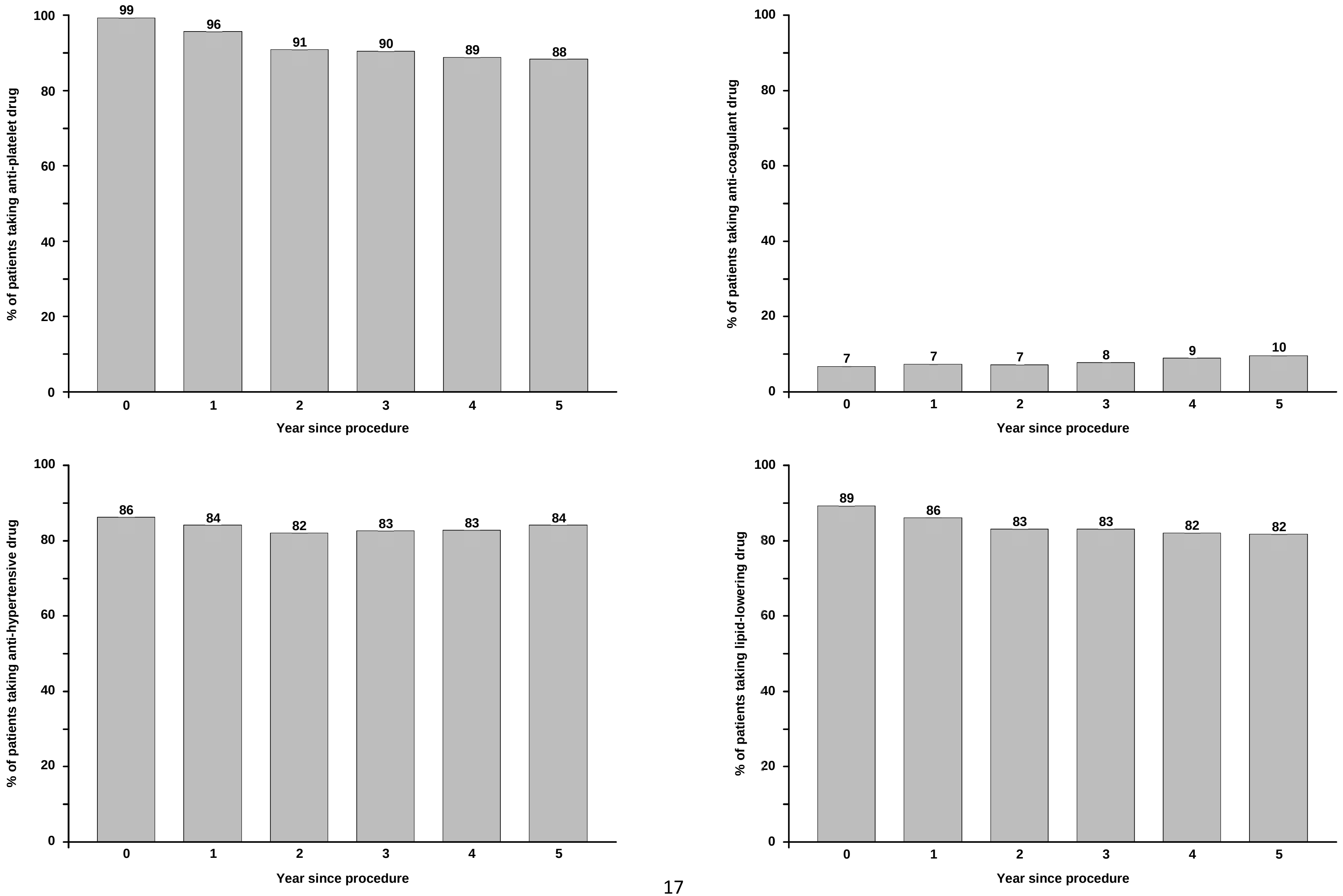

Webfigure W4: Ipsilateral non-procedural strokes, either including or excluding procedural events

(A) Procedural death or disability, or non-procedural ipsilateral fatal or disabling stroke; (B) Non-procedural ipsilateral fatal or disabling stroke; (C) Procedural death or stroke, or non-procedural ipsilateral stroke; (D) Non-procedural ipsilateral stroke

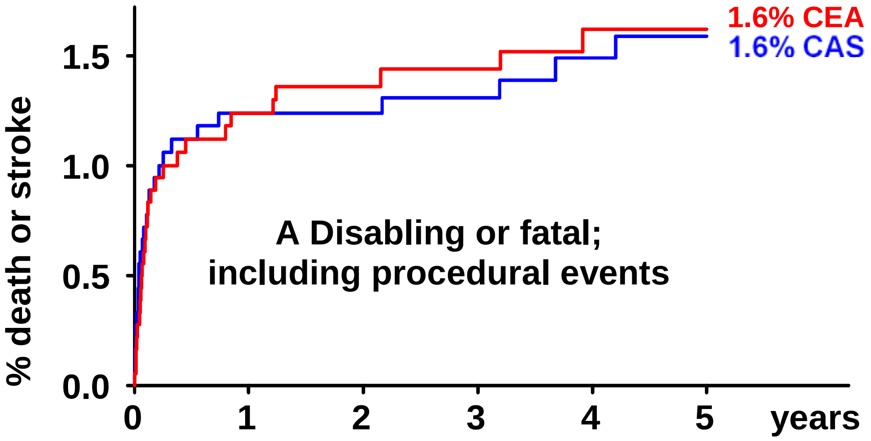

No. at risk (and, in each year, no. of events and annual rate)

CAS	1811	(22, 1.3%)	1639	(0, 0.0%)	1408	(1, 0.1%)	1186	(2, 0.2%)	993	(1, 0.1%)	789 (1, 0.0%)
CEA	1814	(22, 1.3%)	1625	(2, 0.1%)	1422	(1, 0.1%)	1196	(2, 0.2%)	988	(0, 0.0%)	814 (6, 0.3%)

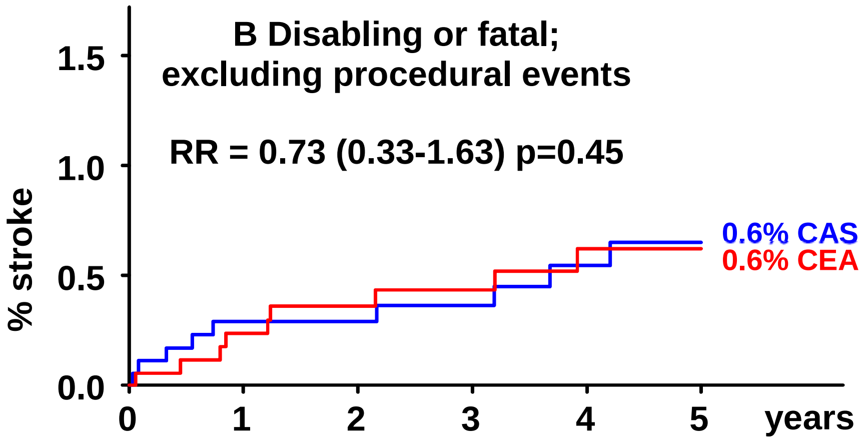

No. at risk (and, in each year, no. of events and annual rate)

CAS	1811	(5, 0.3%)	1639	(0, 0.0%)	1408	(1, 0.1%)	1186	(2, 0.2%)	993	(1, 0.1%)	789 (1, 0.0%)
CEA	1814	(4, 0.2%)	1625	(2, 0.1%)	1422	(1, 0.1%)	1196	(2, 0.2%)	988	(0, 0.0%)	814 (5, 0.2%)

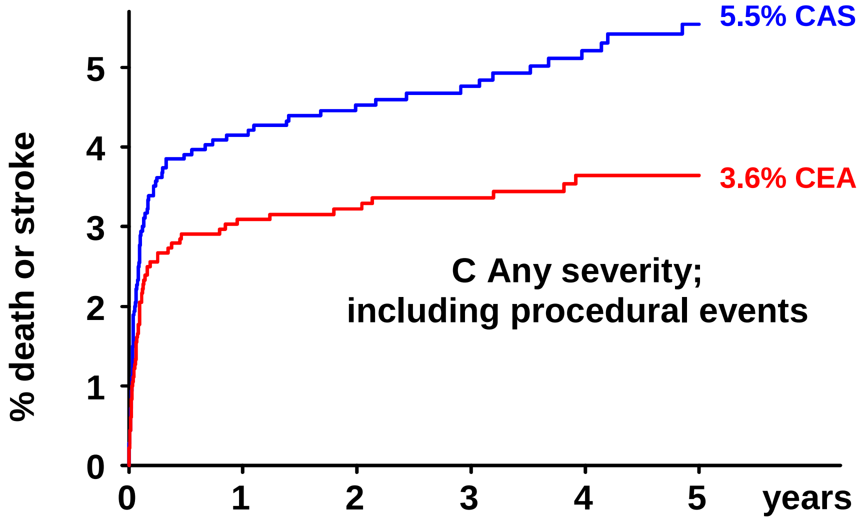

No. at risk (and, in each year, no. of events and annual rate)

CAS	1811	(74, 4.5%)	1594	(6, 0.4%)	1366	(3, 0.2%)	1150	(5, 0.5%)	960	(3, 0.3%)	765 (8, 0.4%)
CEA	1814	(55, 3.3%)	1596	(2, 0.1%)	1398	(2, 0.2%)	1176	(3, 0.3%)	967	(0, 0.0%)	796 (11, 0.5%)

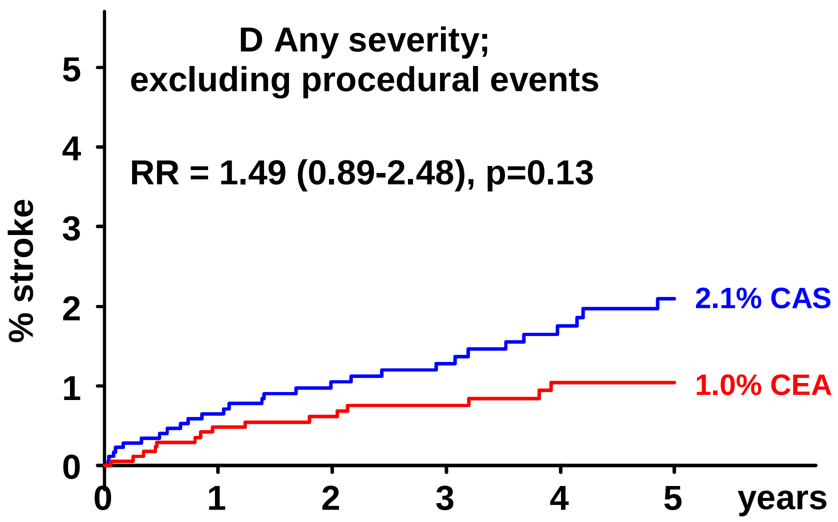

No. at risk (and, in each year, no. of events and annual rate)

CAS	1811	(11, 0.7%)	1594	(6, 0.4%)	1366	(3, 0.2%)	1150	(5, 0.5%)	960	(3, 0.3%)	765 (7, 0.3%)
CEA	1814	(8, 0.5%)	1596	(2, 0.1%)	1398	(2, 0.2%)	1176	(3, 0.3%)	967	(0, 0.0%)	796 (9, 0.4%)

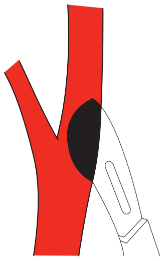

NIHR HTA/BUPA Foundation/University of Oxford

Asymptomatic Carotid Surgery Trial (ACST-2)

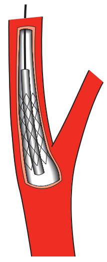

A large, simple randomised trial to compare carotid endarterectomy versus carotid artery stenting to prevent stroke

If a patient needs a procedural intervention for asymptomatic carotid stenosis, there may be substantial uncertainty whether to opt for carotid endarterectomy (CEA) or carotid artery stenting (CAS). ACST-2 seeks to randomise such individuals between CEA and CAS to compare both the immediate hazards of the two procedures when done by experienced doctors, and the subsequent stroke rates over the next 5 to 10 years. ACST-1 (1993-2003) was a trial of CEA versus no immediate procedure (showing CEA could be effective), and involved 3000 patients. Its successor, ACST-2, can succeed only if many thousands of patients are randomised. Hence, the workload per patient is minimised, so that the study can be integrated easily into routine health care. The protocol and all forms are available on www.acst.org.uk

Eligibility: Patient has asymptomatic carotid artery stenosis that is thought to need some procedural intervention; angiography shows CEA and CAS are both anatomically practicable; but, both doctor and patient are **substantially uncertain** whether CEA or CAS is preferable.

Information and consent: If (perhaps even before any magnetic resonance, CT or other angiography) you think a patient might well be eligible, then mention the study to the patient and give the information leaflet for the patient to take away for consideration. The consent section of the information leaflet requires contact details of the patient (for an annual letter from the trial centre) and of the family doctor and 1 or 2 friends or relatives (in case contact is lost).

Randomisation: After consent has been signed, complete at least the first half of the 1-page randomisation form before calling the 24-hour randomisation number +44 (0)18 65 76 56 15 to obtain the treatment allocation (CEA or CAS) and the 6-digit patient ID. This call takes about 2 minutes. Plan for the allocated procedure (CEA or CAS) to be done soon.

Treatment and 1-month post-procedural follow-up: The allocated procedure must be done by a collaborator whose Track Record for that procedure has been approved. Review the patient 1 month afterwards and complete a short form to describe carotid patency and any peri- or post-procedural events.

Long-term follow-up: Annual follow-up for at least 5 years (to monitor any strokes) will be by the ACST office writing to the patient. After the 1-month post-procedural form, no further follow-up by the doctor is required (unless fuller details of a self-reported stroke need to be provided).

As the study is so easy, many hundreds of doctors and many thousands of patients can take part, and uniquely reliable evidence will then emerge comparing the immediate and the long-term safety of CEA and CAS. If a few thousand patients are randomised the results will be useful; if several thousand are randomised the results will be more useful; and if really large numbers are randomised then the results could affect the treatment of millions of patients in future decades.

Asymptomatic carotid artery stenosis?

Substantially uncertain whether to treat with carotid endarterectomy or carotid artery stenting?

CONSIDER FOR ACST-2

ACST-2 Protocol: Version 4.2, December 2007 (UK MREC approval no. 05Q0201/66) ISRCTN21144362

ACST/P/2/1207

To enquire about the trial, contact the ACST office:

ACST, Dept. of Cardiac & Vascular Sciences
St George's University of London, SW17 0RE, UK
email: acst@sgul.ac.uk
tel: +44 (0) 20 87 25 37 46
fax: +44 (0) 20 87 25 37 82

or, visit the ACST website: www.acst.org.uk

with downloadable copies of the patient information leaflet (in various languages), this protocol and the study forms

To RANDOMISE a patient, telephone +44 (0) 18 65 76 56 15

ACST-2 PROTOCOL

Contents

Website address, and contents of the ACST-2 ring binder ii
Background 1
Design and Objectives 2
Starting at a centre: approving procedural Track Records 2
Identifying potentially eligible patients 3
Offering (and re-offering) information leaflet and consent form 3
Randomising by telephone 3
Treating (performing the allocated procedure) 3
1-month post-procedural follow-up 4
Long-term annual follow-up (organised by the ACST office) 4
Strokes and other major events 4
Economic evaluation 5
Sample size, data analysis and safety monitoring 5
Patient withdrawal 6
Trial organisation 6
Funding 6
Protocol modifications 6
Publication 6
Trial organisation and committees 7
References 8

Appendices

Appendix 1 – Patient information leaflet (8 pages, including consent form) 9
Appendix 2 – Randomisation form 15
Appendix 3 – 1-month post-procedural form 16
Appendix 4 – Patient annual follow-up letter and questionnaire 18
(with alternative follow-up letter to family doctor/friend/relative)
Clinic wall poster 22
Memorandum of intent to collaborate in ACST-2: 23

(Appendices 1& 4 are also available in various other languages on www.acst.org.uk)

Protocol Summary Back cover

This protocol, and printable copies of all forms & leaflets, can be downloaded from www.acst.org.uk

Contents of the ACST ring binder

- Information leaflets (which contain blank consent forms) in the local language(s)
- Randomisation forms (which also provide 2 envelopes and the 1-month follow-up forms)
- A few spare copies of the full protocol (and of protocol summaries)
- Clinic Wall Posters

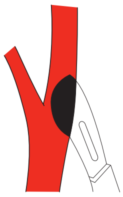

ACST-2

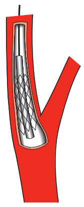

Background

Atherosclerotic narrowing of the carotid arteries can cause stroke, and about 100,000 people in the UK and at least one million people in Europe alone have severe stenosis (narrowing) in one or both of the carotid arteries in their neck[1, 2].

Treatments for patients with carotid artery stenosis

Medical treatment: Appropriate medical treatment with anti-platelet, anti-hypertensive and cholesterol-lowering medicines helps to prevent both heart attack and stroke. In addition, if there is carotid stenosis then non-pharmacological interventional procedures (surgery or stenting) can be used to reduce still further the risk that it will cause a stroke over the next few years.

Carotid endarterectomy (CEA): In 1991 two large trials of surgery (CEA) to remove carotid artery stenosis in 'symptomatic' patients (ie, those who had had a stroke or stroke-like symptoms within the last few months, irrespective of whether any symptoms still persisted) showed that CEA reduced the risk of future stroke from that stenosis[3,4]. CEA is now widely used for stroke prevention in symptomatic patients. The first Asymptomatic Carotid Surgery Trial (ACST-1)[5] and a parallel trial in North America[6] then investigated the role of CEA in a total of 5000 patients with carotid stenosis, but with no stroke or stroke-like symptoms during the previous 6 months. In ACST-1 3000 patients were randomised between medical treatment only or medical treatment and 'immediate' surgery. CEA involved a small (~3%) but definite peri-procedural risk of stroke or death, a substantial (~3% vs ~12%) reduction in the subsequent stroke rate over the next 5 years and hence a net reduction (~6% vs ~12%) in the overall 5-year risk of stroke or peri-procedural death. The 5-year findings of ACST-1 are already changing surgical practice, and long-term follow-up of stroke rates continues[7].

Carotid artery stenting (CAS): CAS is a newer method of treating carotid stenosis, usually via a distant artery, without carotid surgery. If the procedure starts from the groin then a catheter is passed from there up the femoral artery, up the aorta and then into the narrowed carotid artery, and a wire mesh stent is passed up the catheter and placed across the narrowed portion of the carotid artery. A balloon can then be inflated inside the stent to widen it and keep the artery open. The catheter and balloon are then removed. During stent placement some of the diseased artery may crumble, blocking the blood supply to some large or small part of the brain and causing a major or minor stroke. Compared with CEA, CAS avoids surgical wound discomfort, is usually performed under local anaesthetic, could shorten hospital stay, might reduce the risk of peri-procedural heart attack or stroke and may be more acceptable to the patient than surgery. There is, however, substantial uncertainty about the immediate hazards and long-term reliability of CAS, compared to CEA, when both are done by experienced doctors.

The need for a large-scale randomised trial comparing CEA vs CAS: A Cochrane meta-analysis of CEA vs CAS trials (mainly in symptomatic patients) stated that 'the current evidence does not support a widespread change in clinical practice away from recommending CEA as the treatment of choice for suitable carotid artery stenosis. There is a strong case to continue recruitment in the current randomised trials comparing carotid stenting with [versus] endarterectomy'[8]. Multicentre trials, undertaken mainly in symptomatic patients (eg, ICSS, SPACE, EVA-3S, CREST & SAPPHIRE[9-13]), have not yet resolved this uncertainty, and will probably be of limited size. Much larger trials are now needed, particularly in asymptomatic patients. The European Stroke Initiative recommendations for stroke management supported this, and stated that 'carotid angioplasty (balloon dilatation), with or without stenting, is not routinely recommended for patients with asymptomatic carotid stenosis. It may be considered in the context of randomised clinical trials'[14].

Design and objectives

ACST-2 is a large, simple, randomised trial of CEA versus CAS for stroke prevention, and is designed to maximise recruitment by minimising each collaborator's workload. It can be integrated easily into routine health care, as minimal information is required at randomisation and at the 1-month follow-up after the procedure (CEA or CAS). Annual follow-up will then be organised by the ACST office. The randomisation form and 1-month post-procedural form are the ONLY forms that routinely need completion by the doctor. The trial will be international and will randomise patients with asymptomatic carotid artery stenosis in whom prompt physical intervention is thought to be needed, but where (even after magnetic resonance [MRA], computerised tomography [CTA] or some other type of angiography has shown both CEA and CAS to be anatomically practicable) there is still **substantial uncertainty** shared by patient and doctor about whether CEA or CAS is the more appropriate choice. Half of the patients will be randomised to CEA and half to CAS, then all are to be followed up for at least 5 years (mainly by post) and analysed on an intention-to-treat basis. Basing eligibility on uncertainty should ensure large-scale recruitment of an appropriately heterogeneous group. This increases the medical value of the study, perhaps making it possible to determine whether the net effects of CEA/CAS are influenced by certain patient characteristics recorded at entry.

Primary objectives: To compare 1) peri-procedural risks (myocardial infarction [MI], stroke and death within the first month after the allocated CEA or CAS is attempted by an experienced practitioner), and 2) long-term (up to 5 or more years) prevention of stroke, particularly disabling or fatal stroke, in subsequent years.

Secondary objectives: Depending on numbers eventually randomised, the data may enable some types of patients to be identified in which one or other procedure is clearly preferable. As part of a health economic evaluation, procedural costs and stroke-related healthcare costs and quality of life will be assessed.

Starting at a centre: approving procedural Track Records

Local collaborators: Each centre must have a collaborating neurologist (or stroke physician), vascular surgeon and stenting interventionalist. They will be jointly responsible for patient recruitment, treatment and follow-up. The stenting interventionalist can be a radiologist, cardiologist, surgeon or physician with specialist training in carotid stenting. A 'centre' can be organised between colleagues in neighbouring hospitals, **as long as locally practicable arrangements can be made to ensure that the information leaflet (Appendix 1) will be offered to many potentially eligible patients in good time for randomisation to be properly considered** (ie, before one or the other procedure has already been effectively selected). For this, collaboration could be sought with all local centres that do carotid ultrasound, so that the trial information leaflet can be offered as soon as possible after stenosis has been found.

Approval of Track Records: Vascular surgeons who may perform CEA in the trial should already have had a reasonable amount of successful experience with the procedure. Likewise, interventionalists who may perform CAS in the trial should already have had a reasonable amount of experience with up-to-date techniques of stenting. Hence, before the trial is started at a centre, each collaborator who may perform trial procedures should send a 'Track Record' of their previous experience with CEA or CAS (as appropriate) to the ACST office (perhaps using the downloadable form on www.acst.org.uk). This record should be countersigned (as having been seen) by the local collaborating stroke physician or neurologist. It will document details of the last 25, 50 or 100 procedures attempted (depending upon experience): range of dates when attempted (which, for CAS, should include at least 25 patients done using modern materials within the last few years); description of (and comments on any special reasons for) any technical failures; numbers of symptomatic and of asymptomatic patients; and, for both, number of strokes (fatal or non-fatal) and non-stroke deaths within 1 month of the procedure.

These records will be anonymised and then reviewed by the technical management committee. If there is not yet enough successful experience by the surgeon or by the stenting interventionalist at the centre then the start of the trial at that centre will be postponed until there is. In general (except for any cases where there were special reasons for technical failure), collaborators should have ≤8% stroke and death risk for symptomatic patients and ≤4% stroke and death risk for asymptomatic patients, as in previous major trials,[3-6] or some appropriate combination of these percentages.

Ethical approval is required for each centre using this protocol, and the ACST office will help prospective collaborators with the process of obtaining this (to minimise the time taken). In parallel with this, a 'Memorandum of intent' to collaborate (a standard version of which is on page 23 and is also on the website) has to be signed at each centre, and countersigned by the University of Oxford. Once this has been done, ethical approval has been obtained and both Track Records (for CEA and for CAS) approved centrally, eligible patients can be enrolled.

Identifying potentially eligible patients

Potential eligibility (and information leaflet)

- Carotid artery stenosis detectable by duplex ultrasound, with no ipsilateral carotid territory symptoms (or none for some months) and no previous procedure done on it, which might well need procedural treatment now with CEA or CAS. **The information leaflet can be offered even before this is certain**
- Already started any appropriate medical treatment (eg, statin, aspirin etc), and already recovered from any necessary coronary procedures (eg, CABG)
- Patient seems fit and willing for follow-up in person (at 1 month) and by annual letter (for at least 5 years)

Investigations show that both procedures (CEA and CAS) appear to be practicable and appropriate

- Some type of angiography (eg, MRA or CTA) has already been done that has shown that CEA and CAS would both be anatomically practicable. **The information leaflet can then be offered (or re-offered)**
- Doctor and (after information) patient both **substantially uncertain** about whether to treat with CEA or CAS, and the doctor sees no clear indication/contra-indication for either procedure

Contra-indications are specified not by the protocol but by the doctor, and might include:

- Small likelihood of worthwhile benefit (eg, very low risk of stroke because stenosis is very minor, or major co-morbidity or life-threatening disease, such as advanced cancer)
- Unsuitable for one or other procedure (eg, stenosis at carotid siphon that is inaccessible for CEA, or complex vasculature below the stenosis that would hinder CAS, or patient unfit for major surgery)

Offering information leaflet and consent form

- As soon as possible after stenosis has been diagnosed, the possibility of joining the trial should be mentioned to the patient, who can then be given the information leaflet (Appendix 1) to consider. This can be done even before any angiography has been undertaken, when it is still not clear whether the patient will be eligible. Patients may wish to take the information leaflet away before deciding whether they are likely to join the study
- The information leaflet can also be offered (or re-offered) later, without pressure. If the patient is found to be eligible and does decide to join, then written informed consent will be needed, and the patient should understand that they will be contacted annually by an ACST office (probably by post) for at least 5 years
- When signing the consent section of the information leaflet, the patient will be asked to give contact details of the family doctor, and of 1 or 2 friends or relatives (with their agreement), any of whom could be contacted if the patient cannot be traced by the ACST office. (Patients who take the information leaflet away are asked to bring these contact details to the next clinic visit, to avoid delay, but clinic staff may still need to help get them completed)

Randomising by telephone

- Complete at least part 1 of the randomisation form (Appendix 2) before telephoning to enter the patient, as these details are needed in the phone call. (The rest can be done later.)
- Telephone the randomisation service on +44 (0)18 65 76 56 15. The randomising collaborator will be asked to confirm that the consent section of the information leaflet (with contact details) has already been signed, and to answer the questions on the first half of the randomisation notepad
- The collaborator will then be given a unique 6-digit patient identification number and the treatment allocation to CEA or CAS. **The patient is now in ACST-2**. The ID number and allocation should be written onto the randomisation form and onto the foot of the consent form
- Arrange for the allocated procedure (CEA or CAS) to be done as soon as possible (by a collaborator whose Track Record for that procedure has been approved), and send off completed randomisation & consent forms
- Complete remainder of **Randomisation Form** and send, with signed **Consent Form,** to the study office

Treating (performing the allocated procedure)

- The CEA or CAS should be done as soon as possible (ideally within the first month after randomisation) by a collaborator whose Track Record for that procedure has been approved. It is the responsibility of this collaborator to use techniques and equipment that are appropriate for routine clinical practice (eg, in Europe, CE-marked stents). Cerebral protection devices are optional (ie, at the collaborator's discretion)
- **Before** discharge:
    - Schedule duplex ultrasound and a 1-month clinical follow-up visit
    - Schedule assessment by a neurologist/stroke physician (perhaps at a return visit within one month of the procedure) of whether or not the patient had a peri-procedural stroke or MI
- All other care remains the responsibility of the patient's doctor, and not the trial. Patients do not need to undergo any other tests or examinations beyond those provided as part of their routine care

1-month post-procedural follow-up

- The 1-month post-procedural form (Appendix 3) should be completed and sent to the ACST office
- If the 1-month post-procedural follow-up visit is missed, seek another appointment until contact is made (or the information is obtained otherwise). Even if the patient undergoes another procedure instead of the allocated procedure, the 1-month post-procedural form should still be completed
- If it is decided that no procedure will be undertaken, please write to the trial office and explain why

Long-term annual follow-up (organised by the ACST office)

- Patients will be contacted annually for at least 5 years by a letter originating from the international ACST office asking if they remain well, and enclosing a brief questionnaire (Appendix 4). Both will be in the patient's own language, with a prepaid envelope for return to an ACST office
- If the patient replies that they have had a stroke, an appropriate doctor will be contacted to seek details of the stroke
- If the patient does not reply, a similar letter (Appendix 5) will be sent to the family doctor, or to any friends or relatives whose contact details were given when the patient joined the study
- If the patient is too disabled to complete the questionnaire, someone else (eg, the patient's carer) can complete it with answers provided by the patient, or can complete it based on their own assessment of the patient
- In the few cases where the patient and the contacts they gave do not provide the annual information, the local collaborator or country collaborator office may be asked to help trace the patient. UK patients will be flagged with the Office For National Statistics upon entry into the trial and, where available, national data repositories in other countries will likewise be used to facilitate data collection during the study

Although long-term follow-up will be organised by the ACST office, if a collaborator happens to know that a trial patient has had a stroke or has died, please write to the ACST office, describing if possible the nature and severity of the stroke or stating whether death was due to stroke.

Strokes and other major events

Strokes (within the first post-procedural month or during long-term postal follow-up)

Stroke outcome will be classified using the modified Rankin disability scale:

0 No symptoms at all from the stroke
1 No significant disability, despite any symptoms from the stroke: able to carry out usual activities
2 Slight disability because of the stroke: unable to carry out all previous activities but able to look after own affairs without assistance
3 Moderate disability from the stroke: requiring some help, but able to walk without assistance
4 Moderately severe disability from the stroke: unable to walk without assistance and unable to attend to own bodily needs without assistance
5 Severe disability from it: bedridden, incontinent and requiring constant nursing care and attention
6 Died directly or indirectly from the stroke

Peri- or post-procedural myocardial infarction (only within the first month)

It is necessary to report MI only if this occurs during the peri- or post-procedural (1 month) period. If more than one MI occurs in this month, each should be reported. A definite diagnosis of MI can be made only if 2 of the following criteria are fulfilled:

1 Symptoms consistent with MI
2 Positive enzyme or biomarker (eg, troponin-T) changes consistent with MI
3 ECG changes consistent with MI

Death (within the first month, or later)

If the patient dies within one month of the trial procedure (CEA/CAS), the cause and circumstances should be described on the 1-month post-procedural form. Otherwise, follow-up will be by post from the ACST office, and the only information that is required on the death of a patient is the date of death and whether or not the cause of death was related to stroke. (This will, in general, be obtainable by the ACST office without any involvement of the local collaborators.)

Economic evaluation

The costs of the trial interventions and of any stroke-related impairment of quality of life will be evaluated, but this will not involve the local participating doctors. For all UK patients, direct access will be sought to the NHS electronic records of hospital activity. (These give, for each NHS hospital admission during the years of follow-up, the main reasons for the admission, the procedures undertaken, the duration of stay and the discharge diagnosis.) This allows NHS resource use during the entire follow-up period to be calculated, and the proportion attributed to stroke to be estimated. In addition, for all UK patients who have a peri-procedural MI or stroke (plus a matched sample of those who do not) a self-completion quality of life questionnaire (EuroQOL EQ-5D) will be sent directly from the trial office. Finally, every patient who has a stroke at any time during the study will be asked annually how it is affecting them.

Resource use during the treatment and follow-up periods will be estimated. The main components will be (a) the initial procedural costs; (b) further short-term re-treatment costs (ie, repeat or further procedures within a month); (c) the costs of any MIs within the first month and any stroke costs (both for strokes caused by the trial procedures and for those considered not to have been). The length of stay in hospital will be collected for these events. Annual follow-up questionnaires sent by the central trial office directly to the patient will collect data on the level of care currently required for a particular stroke patient (modified Rankin score), as well as information on how long the patient has had to have hospital or nursing home care for it. It will also seek information about strokes or carotid procedures after the first month. (Standard costs will be assumed for these procedures.) The economic analyses will evaluate the stroke-related quality of life at one month after the trial procedures as well as short and longer-term stroke outcome and costs.

Sample size, data analysis and safety monitoring

As the study is so easy, many hundreds of doctors and many thousands of patients can take part, and uniquely reliable evidence will then emerge comparing the immediate and the long-term safety of CEA and CAS. If a few thousand patients are randomised the results will be useful; if several thousand are randomised the results will be more useful; and if really large numbers are randomised then the results could affect the treatment of millions of patients in future decades.

The main outcomes will be MI, stroke or death 1 month after the allocated procedure (CEA or CAS), and long-term (up to 5 or more years) stroke rates. With 5000 randomised, a decrease of about 60% in the peri-procedural myocardial infarction rate with stenting versus surgery (eg, 2% CEA vs 0.8% CAS) and an increase of about 60% in the 5-year stroke rate (eg, 3% CEA vs 5% CAS) could both be detected at $P<0.001$ with 80% probability (ie, with 80% statistical power), or at $2P<0.05$ with 95% power. The exact magnitude of any effect is currently not known, hence the need for the trial, but, taking into account existing information from other trials of CAS vs CEA, effects of this size might be realistic, meaningful and worthwhile. Even smaller effects could be of substantial interest, but might require much larger numbers to be studied.

The results will be displayed using Kaplan-Meier graphs. Logrank analyses will compare stroke rates between those allocated CEA and those allocated CAS in specific time periods. All patients should be followed up (unless they choose to withdraw from follow-up) whether the trial procedure is carried out or not, since the main trial analyses will be on an 'intention-to-treat' basis. By the end of the recruitment period, data will be available to consider the peri-procedural outcomes, and with (by then) about 2 years follow-up on average, analyses will be possible of the early effects of CEA vs CAS on the annual incidence of various types of stroke, disabling stroke and fatal stroke. Continued follow-up will allow more powerful analyses of these longer-term outcomes.

Subgroup analyses will be undertaken, where appropriate, to assess the relevance of prognostic factors. In ACST-1[5], the 5-year risk of non-peri-procedural carotid territory ischaemic stroke was analysed in a number of categories, defined by: stroke severity; age; sex; pre-randomisation cholesterol; pre-randomisation blood pressure; ipsilateral and contralateral carotid artery diameter reduction – ie, degree of stenosis; plaque echolucency; ipsilateral and contralateral carotid territory status at entry; and diabetes and other problems, as recorded at entry. Similar analyses are planned in ACST-2.

Data monitoring committee: During the study, interim analyses of major events will be supplied at least annually to an independent Data Monitoring Committee (DMC). The DMC will advise the Trial Steering Committee (TSC) whether there is an unacceptably high morbidity associated with CEA or CAS (either overall, or in particular centres, or in the centres with more limited prior experience), or if there is clear evidence that, for all patients or some particular types of patient, there is proof beyond reasonable doubt that one or the other procedure is preferable. Until then, the TSC and collaborators will otherwise remain ignorant of interim results.

Appropriate criteria of proof beyond reasonable doubt cannot be specified precisely, but a difference of at least 3 standard deviations in an interim analysis of a major endpoint may be needed to justify halting or modifying such a study prematurely. If this criterion were to be adopted, it would have the practical advantage that the exact number of interim analyses would be of little importance, so no fixed schedule is proposed.

At any point, anyone associated with the study may write through the ACST office to the DMC Chair drawing attention to any concerns they may have about the possibility of particular side-effects, or of particular categories of patient requiring special study, or indeed about any other matters thought relevant.

Patient withdrawal

A few of those who originally agreed to join the study may later change their minds and withdraw, but this should not materially affect the scientific integrity of the study. Some such patients may still be willing for information about their health to be collected according to the trial protocol but may prefer not to be contacted directly. Others may wish to withdraw entirely. In either case, if, after agreeing to join, the patient subsequently changes his or her mind, (s)he is free to do so without this adversely affecting his/her medical care. Similarly, the patient's doctor is free to give any other treatment that is considered to be in the patient's best interest.

Trial organisation

The study will be managed on a day-to-day basis by the ACST office based at St George's University of London. All enquiries about the study should be directed to this office. Randomisation will be through the Clinical Trial Service Unit (CTSU), Oxford. Data will be stored on secure UK University computers, with identifiers held separately. The trial will be governed by a project management group that is responsible to the mainly independent Trial Steering Committee (TSC). A Technical Management Committee will be responsible for approving interventionalists/surgeons wishing to participate, after reviewing their procedural Track Records. An Endpoint Review Committee will classify strokes, and any other relevant outcomes. An Economic Evaluation Committee will guide the principal investigators on the UK and other health economic implications. Finally, the independent Data Monitoring Committee (DMC) will undertake interim analyses of trial data.

Funding

The UK National Institute of Health Research (NIHR) Health Technology Assessment programme has contributed towards the first 5 years of the trial and the BUPA Foundation, a UK medical research charity, is contributing towards at least the first 3 years. In addition the University of Oxford's Clinical Trial Service Unit (CTSU) has provided some assistance free of charge. Doctors and patients who participate in the study are not paid to do so, and the final results will be freely available on the website and in journals.

Protocol modifications

This protocol, originally prepared by the ACST Office and CTSU, has been extensively reviewed and approved (MREC approval no. 05Q0201/66). It should not be modified unless this is essential. If any modification appears to be needed to comply with national or local regulations, it must be discussed in advance with the ACST office.

Publication

Results of the study will be prepared by a writing committee and circulated to all collaborators for comments prior to publication, Results will be published in the name of the ACST collaborative group. The chief acknowledgement will be to the patients who participate in the study.

Trial organisation and committees

Principal Investigators (PIs)

Alison Halliday, Professor of Vascular Surgical Studies, St George's University of London (SGUL), UK

Christina Davies, Senior Research Fellow, CTSU, Oxford, UK

Richard Peto, Professor of Medical Statistics & Epidemiology, CTSU, Oxford, UK

Jean-Pierre Becquemin, Professor of Vascular Surgery, Hôpital Henri Mondor, Paris, France

Alastair Gray, Director, Health Economics Research Centre (HERC), Oxford, UK

Borislava Mihaylova, HERC, Oxford, UK

Trial Steering Committee (TSC), reporting to (but independent of) the funding bodies

John Potter (chair), Professor of Medicine for the Elderly, University of East Anglia, UK

Christina Davies (co-PI)

Marcus Flather, Clinical Trials & Evaluation Unit, Royal Brompton Hospital, London, UK

Alison Halliday (PI)

Sumaira Macdonald, Consultant Radiologist, Freeman Hospital, Newcastle, UK

Averil Mansfield, Chair, Stroke Association, UK

Richard Peto (co-PI)

David Simpson, Lay Patient Representative, Oxford, UK

Dafydd Thomas, National Hospital for Neurology & Neurosurgery, London, UK

Technical Management Committee (reviewing Track Records of prospective collaborators)

Michael Gough (chair), Consultant Vascular Surgeon, Leeds, UK

Marc Bosiers, Head of Surgery, Hôpital A.Z St. Blasius, Dendermonde, Belgium

Piergiorgio Cao, Professor, Istituto di Clinica Chirugica Generalo, Perugia, Italy

Sumaira Macdonald (TSC; interventional radiologist)

Endpoint Review Committee (classifying the nature and severity of any strokes)

Peter Rothwell (chair), Professor of Clinical Neurology, University of Oxford, UK.

Peter Leopold, Consultant Vascular Surgeon, Frimley Park Hospital, Surrey, UK

Anna Belli, Consultant Radiologist, SGUL, London, UK

Economic Evaluation Committee

Alastair Gray (chair), HERC, Oxford, UK (co-PI)

Borislava Mihaylova, HERC, Oxford, UK (co-PI)

Frank Vermassen, Department of Vascular Surgery, Ghent University Hospital, Belgium

Jonathan Michaels, Department of Vascular Surgery, University of Sheffield, UK

Independent Data Monitoring Committee (DMC)

Peter Sandercock (chair), Professor of Neurology, University of Edinburgh, UK

Richard Gray, Professor of Medical Statistics, University of Birmingham Clinical Trials Unit, Birmingham, UK

Cliff Shearman, Professor of Vascular Surgery, University of Southampton, UK

Andrew Molyneux, Consultant Neuroradiologist, Frenchay Hospital, Bristol, UK

Project Management Group

All PIs and other individuals named above, except DMC members

In attendance: project staff from the ACST office, SGUL (2007: Elizabeth Hayter, Karen Phekoo) and the Oxford CTSU (2007: Mike Lay, Andrew Munday, Alan Young)

References

1. Lopez AD, Mathers CD, Ezzati M, Jamison DT, Murray CJL (Eds). Global Burden of Disease & Risk Factors. Oxford University Press and The World Bank, Washington DC, 2006.
2. Berry E, Kelly S, Westwood ME, Davies LM, Gough MJ et al. The cost-effectiveness of magnetic resonance angiography for carotid artery stenosis and peripheral vascular disease: a systematic review. Health Technology Assessment 2002; 6: no. 7.
3. European Carotid Surgery Trialists' Collaborative Group. MRC European Carotid Surgery Trial: interim results for symptomatic patients with severe (70-99%) or with mild (0-29%) carotid stenosis. Lancet 1991; 337: 1235-43.
4. North American Symptomatic Carotid Endarterectomy Trial Collaborators. Beneficial effect of carotid endarterectomy in symptomatic patients with high-grade stenosis. N Engl J Med 1991; 325: 445-53.
5. MRC Asymptomatic Carotid Surgery Trial (ACST) Collaborative Group. Prevention of disabling and fatal strokes by successful carotid endarterectomy in patients without recent neurological symptoms: randomised controlled trial. Lancet 2004; 363: 1491-502.
6. Executive Committee for the Asymptomatic Carotid Atherosclerosis Study. Endarterectomy for asymptomatic carotid stenosis. JAMA 1995; 273: 1421-28.
7. Halliday A. The Asymptomatic Carotid Surgery Trial has changed clinical practice. Pages 56-60 in: Vascular and Endovascular Controversies. Biba Publishing, 2006.
8. Coward LJ, Featherstone RL, Brown MM. Percutaneous transluminal angioplasty and stenting for carotid artery stenosis (Cochrane Review). In: The Cochrane Library, Issue 3, 2004. Chichester, UK: Wiley & Sons.
9. Featherstone RL, Brown MM, Coward LJ, on behalf of the ICSS Investigators. International Carotid Stenting Study: Protocol for a randomised clinical trial comparing carotid stenting with endarterectomy in symptomatic carotid artery stenosis. Cereb Dis 2004; 18: 69-74.
10. The SPACE Collaborative Group. 30 day results from the SPACE trial of stent-protected angioplasty versus carotid endarterectomy in symptomatic patients: a randomised non-inferiority trial. Lancet 2006; 368: 1239-47
11. Mas J-L, Chatellier G, Beyssen B, Branchereau A et al. Endarterectomy versus stenting in patients with symptomatic severe carotid stenosis N Engl J Med 2006; 355: 1660-71.
12. Hobson RW. Carotid artery stenting. Surg Clin North Am 2004; 84: 1281-94.
13. Yadav JS, Wholey MH, Kuntz RE, Fayad P, Katzen BT, Mishkel GJ, Bajwa TK, et al. Protected carotid-artery stenting versus endarterectomy in high-risk patients. N Engl J Med 2004; 351: 1493-501.
14. The European Stroke Initiative Executive Committee and Writing Committee. European Stroke Initiative recommendations for stroke management - update 2003. Cereb Dis 2003; 16: 311-37.

Appendix 1 – Patient information leaflet (front page)

[Also available in various other languages on www.acst.org.uk]

ACST-2 patient information leaflet

Information about a research study that you may be invited to join

Patients who have a narrowing in one of the arteries that takes blood to the brain may need something done to keep that artery open (even if the narrowing has not caused a stroke, or any other symptoms). There are two main ways of doing this (called carotid endartarectomy [CEA] or carotid artery stenting [CAS]), but they cannot both be done at the same time on the same narrowed artery. If (maybe after further tests) your doctor is still **uncertain** which of these two procedures to recommend for you, then you may be invited to join an international study comparing them.

This information leaflet describes that study. Briefly, half the patients who join it get CEA, half get CAS, and after the allocated procedure has been done we send you, once a year for at least 5 years, a short questionnaire asking how you have been. If you are invited to join, and agree to do so, then we would need not only your own name and address but also (in case we lose contact) that of your family doctor and of 1 or 2 friends or relatives – please let them know that you've given us their details. Thank you for taking the time to read this.

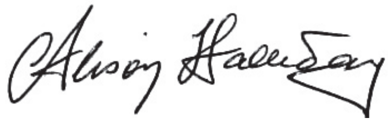

Dr. Alison Halliday, study director
University of London

PS. There are no payments to doctors or patients who join this study, and the eventual results will be freely available to help future patients.

December 2007 ***Main information on inner pages...***

Notes to doctors: Please see back cover

Possibility of joining a large international study comparing 2 stroke prevention procedures (CEA & CAS)

Before you consider whether to join we would like to summarise why this study is being done and what it will involve. Please discuss this if you wish with friends, relatives or your family doctor.

- Your hospital doctor may already have told you that you are at increased risk of having a stroke because you have a narrowing in one or both of your carotid arteries (the arteries in the neck that supply blood to the brain). Although you don't have any symptoms at present, this narrowing may need to be treated promptly to reduce your risk of having a stroke over the next few years.
- The standard procedure ("**carotid endarterectomy**" - CEA) involves surgery, often under a general anaesthetic, to unblock the inside of the narrowed part of the artery in the neck. We know already that this operation involves some immediate risk, but that it does provide long-term protection against the narrowing causing a stroke.
- A newer procedure ("**carotid artery stenting**" - CAS) can now be used instead. This involves inserting a tube inside the narrowed part of the artery to hold it open. CAS avoids operating on the neck, as the tube is inserted via an artery some distance away (usually in the leg), often with only a local anaesthetic. CAS might be safer and as effective as CEA at preventing stroke, but currently there is not enough information to know this reliably.
- Your hospital doctor is at present uncertain which of these two procedures would be better for you. If any further tests leave the doctor still uncertain, and you too are uncertain, then please consider taking part in this research study (involving thousands of patients like you) to help find out which procedure is the safer and more effective at preventing stroke.
- If, on the other hand, you would definitely prefer CEA or would definitely prefer CAS (or would definitely prefer neither) then please do not join the study; just tell your doctors your wishes.

continued on next page...

- Among those who do join the study, half will be allocated CEA, and half will be allocated CAS. Neither you nor your doctor (nor anyone else) will know beforehand which of these two procedures you will be allocated if you join. This will be determined by the play of chance (as if on the toss of a coin) once you join the study and information about you has been put into the study computer. When the procedure (CEA or CAS) has been allocated, your doctor should arrange for you to get it as soon as possible.
- All other aspects of your care will remain the responsibility of your own doctor, and will not be affected by you being in the study. You will be free to withdraw from the study at any time. If you do withdraw, this will not adversely affect your medical care. The patients (and their doctors) who take part in this study are not paid to do so, and participate freely.
- All the information collected about you during the study will be stored securely on UK University computers and kept strictly confidential. Any published reports of the study will not identify you or any other patients, and will be made publicly available on the study website.
- Your doctor will want to see you about 1 month after the procedure has been done to assess your general health. Then the study organisers would like to send you a brief questionnaire once a year for at least 5 years, probably by letter (or by telephone or email), to ask how you are doing.
- If you decide to take part, you will be asked to sign a consent form (on page 7 of this leaflet) saying that you agree to do so, and your family doctor will be sent a letter saying that you have done so.
- On the consent form you will be asked to give the contact details of your family doctor and of 1 or 2 friends or relatives, so we can ask them how you are if we lose contact with you. Please be ready to provide these details if you think you might join the study: see below.

If you might decide to take part, please bring to your next clinic visit contact details of your family doctor and of 1 or 2 friends or relatives (or write them onto the consent form on page 7)

Extra details on next page for patients who want them

ACST-2: Second Asymptomatic Carotid Surgery Trial
Extra details for patients who want them

Background Narrowing in the carotid arteries (the main arteries in the neck that supply blood to the brain), caused by build-up of fatty deposits, is a cause of many strokes. People with this narrowing may be asymptomatic – that is, they may have no symptoms until fragments fall off, lodge in the brain and cause a stroke. The standard procedure to prevent this, "carotid endarterectomy" (CEA), involves operating on the neck to remove the fatty deposits from the artery before they cause stroke-like symptoms or a major stroke. CEA involves some immediate risk but, if successful, provides long-term protection against the narrowing causing a stroke. An alternative procedure is "carotid artery stenting" (CAS), which involves placing a fine wire mesh tube (called a stent) inside the narrowed artery to hold it open. CAS avoids neck surgery, but we do not yet know how it compares with CEA in immediate risks or in long-term benefits, as previous studies comparing these procedures were too small.

What is the study about? ACST-1 (the first asymptomatic carotid surgery trial, 1993-2003) involved 3000 patients, and showed that CEA could be effective. ACST-2 will now involve many thousands of patients where both the patient and doctor are **substantially uncertain** whether to opt for CEA or for the newer procedure, CAS. Half of the patients will be allocated CEA and half CAS to treat the narrowed artery in their neck. The relatively small immediate hazards (mainly heart attack, stroke or death) and the small remaining stroke risks over the next few years after these 2 procedures will be compared, and the type and severity of any strokes that may occur will be assessed. This type of large, long-term study will help find out reliably which is the better treatment for future patients like you.

What does the study involve? If you agree to take part, you will be asked to sign a consent form (on page 7 of this informatiom leaflet) and to give contact details of your family doctor and of two friends or relatives. If for some reason we lose contact with you over the next few years, we can then ask them how you are. You will be allocated to either CEA or CAS: this will be decided randomly and unpredictably by the central computer (as if on the toss of a coin). Your hospital doctor will then arrange for the allocated procedure to be carried out as soon as routinely possible. If after joining the study you later change your mind, then you are free to do so without needing to give any reason and without adversely affecting other aspects of your care. Your doctors will continue to see you as normal regardless of whether or not you join (or stay in) the study.

What do the different procedures involve? Whichever operation you have, the doctor treating you will be experienced in the technique and will carry it out according to the usual methods used in your hospital. If you have CEA, you may have a general anaesthetic, and you may have to stay in hospital for several days after surgery. If you have CAS, you will usually have a local anaesthetic and may well be able to go home the following day. If after you join the study your doctor decides for any reason that the allocated procedure no longer seems appropriate, you will be offered the other treatment option if it seems appropriate. This is up to your doctor, and is not controlled by the study.

What will happen after the procedure? You will be seen by a hospital doctor about 1 month after the procedure to assess your general health. Then, every year for at least 5 years, the study organisers would like to send you a short questionnaire about whether you have had any problems possibly linked to your carotid artery (eg, whether you have had a stroke, and, if so, how this has affected you). Your normal medical care should not be affected by your participation in the study.

Are there any risks? Successful treatment of carotid narrowing will reduce the chance of you having a stroke from it in the future, but CEA and CAS themselves carry a relatively small risk of causing an immediate stroke or heart attack. But, your doctor would put you forward for these procedures only if your doctor thinks that, for you, the expected benefits are greater than the risks.

What if something goes wrong? In the event of you being harmed as a result of taking part in this research project, you will retain the same rights of care as any other patient, including access to the usual complaints mechanisms if something was done wrongly. Whilst there are no special compensation arrangements for participants, if you are seriously harmed due to someone's negligence then you would, of course, have the usual grounds for taking legal action. You would receive the appropriate investigations, treatments and care, just like any other non-study patient.

Who is organising the study? The study is organised by the ACST office at St George's University of London, working with the Clinical Trial Service Unit at the University of Oxford (the official sponsor of the study), and the running costs, for at least the first few years, are paid jointly by the UK government's Health Technology Assessment Programme and a UK medical research charity, the BUPA Foundation. The hundreds of doctors and thousands of patients who participate in the study are not paid to do so (so you personally will gain nothing from joining), but the final results will be freely available to help future patients.

When will it provide answers? It will take some years to enrol enough patients to make the study large enough to be reliable, and these patients will then have to be followed up for some years after their treatment to compare the long-term effects of CEA and CAS. While the study is in progress its early findings (and any other new relevant information) will be continually monitored to ensure that the study remains appropriately safe and viable. Long after you join, the final results will be freely available on the study website and published in a scientific medical journal, but neither you nor other patients will be identified when this happens.

Confidentiality We want to collect only the information that is required to help compare CEA with CAS (although we may find we can also use this information for other medical research to help future patients). The information will be treated in strict confidence, held by the study organisers on secure databases on UK University computers, and retained for a minimum of 15 years. Your name, address and date of birth may be passed confidentially to a national records office to help us remain in touch with you, and your medical records may be inspected confidentially by trial regulators and other properly authorised persons to check that we are doing the study properly. Otherwise, any information released outside the trials office will not identify you.

If anything is not clear, or you would like more information, please ask the doctor who gave you this leaflet or your family doctor, or another doctor (eg, the one named on the consent form); or, see www.acst.org.uk

Page 5 of 8

Appendix 1 – Patient consent form (page 7 of patient information leaflet)

[Pages 2 & 6 of the Patient consent form (not shown in the protocol) just say "If you do eventually decide to join, then the consent form on page 7 is what you will be asked to sign. On it are the contact details that you will be asked for. You will be offered this information leaflet to keep (with a copy of the completed and signed consent form as page 7 of it)."]

Consent to join ACST-2, a large international study comparing 2 stroke prevention procedures

- I have read and understood the ACST-2 information leaflet (dated June 2007) and have had the opportunity to ask any questions I want. I understand that if I join there is an equal likelihood that I could be allocated either CEA or CAS.
- I agree that the study organisers can contact me by post (or, perhaps, by telephone or email) for at least 5 years to find out whether I have had a stroke and, if so, how it affects me. If necessary, they can contact my family doctor, or any friends or relatives I name below (with their agreement), for this purpose.
- I agree that my hospital and other records, including this consent form and my family doctor records, may be looked at in confidence by authorised individuals from the study, by Oxford University (the study sponsor) and by regulatory authorities (to check the study is being carried out correctly).
- I understand that national records (including, in the UK, information held by the NHS) may be used to help keep in touch with me or to help find out about any strokes (and that for this purpose my details may be sent, in confidence, to national record offices).

I confirm the above statements, and I agree to take part in this study.

My continued participation is, however, voluntary.
I will be free to withdraw at any time, without giving any reason and without my medical care or legal rights being affected.

______________________	____/____/____	______________________
Name of patient (please PRINT),	date (day/month/year),	and signature.
______________________	____/____/____	______________________
Name of person countersigning consent (PRINT),	date (day/month/year),	and counter-signature.

Name and contact details of a local ACST collaborator (please PRINT):			
**Contact details for an annual letter to find out how you have been (please PRINT)**			
Patient name:		Family doctor:	
Address:		Address:	
Telephone:		Telephone:	
& email (if known):		& email (if known):	
**Contact details of 1 or 2 friends or relatives who can be written to for the annual information if we lose contact with you (please PRINT)**			
Friend/relative (1):		Friend/relative (2):	
Address:		Address:	
Telephone:		Telephone:	
& email (if known):		& email (if known):	

Keep copy of page 7 in hospital notes, give pages 1 - 8 to patient and fax/post original page 7 to ACST Office, Dept of Cardiac & Vascular Sciences, St George's University of London, SW17 0RE, UK (fax +44 (0) 20 87 25 37 82)

To be completed later – ID: ☐-☐-☐ & allocated procedure (CEA/CAS): ☐

ACST/C/1/1207

Page 7 of 8

ACST-2 patient information leaflet (last page) with consent form on previous page

Notes to doctors

- This leaflet can generally be given even before it has been decided whether any carotid procedure will be needed, as soon as significant carotid artery narrowing has been detected (as long as this has caused no recent symptoms).
- Alternatively, it can be given (or re-offered) a little later, after it has been decided that some procedure (CEA/CAS) should be recommended.
- Likewise, the leaflet can be given either before detailed arterial investigation (by MRA or CTA) has checked whether CEA or CAS are both anatomically practicable, or it can be given (or re-offered) afterwards.
- **Please check** that the name of a local ACST collaborator has been written onto the middle of the consent form (page 7 of this leaflet) before giving the leaflet.

ACST Office, Department of Cardiac & Vascular Sciences
St George's University of London, SW17 0RE, UK
(fax +44 (0) 20 87 25 37 82)

Appendix 2 – Randomisation form (3-page fold-out; open once to see randomisation form and envelopes; open again to see 1-month follow-up form)

ACST-2 RANDOMISATION FORM: complete top half (PART 1), then phone randomisation service +44 (0) 18 65 76 56 15 & provide the information in Part 1

☐ Which country are you in?

☐ ACST-2 code for your hospital (If unknown, give hospital name, city & country and your code will be provided)

☐ Name of randomising doctor (**PRINT**)

☐ Family name(s) of patient (**PRINT**)

☐ Main given name(s) of patient (**PRINT**)

d d / m m / y y Date of birth (day/month/year)

☐ Sex (M = male, F = female)

☐ Consent signed? (ie, consent form **already** signed, **with** contact details on it)
Y = YES, N = NO: **MUST** be YES

☐ Angiogram OK? (ie, anatomically suitable by CTA, MRA or other angiogram **both** for CEA **and** for CAS)
Y = YES, N = NO: **MUST** be YES

☐ Side? (Laterality of artery for randomisation, L = Left, R = Right: patients cannot be re-randomised)

☐ Doppler % stenosis? (% stenosis on this side, by duplex doppler)

☐ Echolucent? (Plaque >25% echolucent, Y/N or X = not known)

☐ Contra-lateral stenosis? (%, by duplex doppler)

☐ AF? (Known atrial fibrillation, Y/N)

☐ Diabetic? (On drug or insulin therapy for diabetes, Y/N)

☐ Systolic? (Systolic blood pressure, mmHg)

☐ Diastolic? (Diastolic blood pressure, mmHg)

At the end of the phone call write down ☐ – ☐ – ☐ 6-digit patient ID number (from phone service) and procedure allocated by randomisation ☐ (CEA or CAS)

→ Plan for the allocated procedure (CEA/CAS) to be done soon

Please also enter 6-digit patient ID and allocated procedure onto the foot of the consent form

PART 2: Clinical data *(not asked by telephone; can be completed a little later)*

Left	Right	Data on both left and right carotid territories
☐	☐	Infarct on CT scan in the carotid territory? Y/N/X (X = not done)
☐	☐	Infarct on MRI scan in the carotid territory? Y/N/X (X = not done)
☐	☐	Ever symptomatic in the carotid territory? 0 or N = never, 1 = A. fugax only, 2 = TIA, 3 = stroke

Other clinical data

☐ CAD? (Definite history of coronary artery disease, Y/N)

☐ Renal impairment? (Y/N)

☐ On anti-platelet therapy? (Y/N)

☐ On anti-coagulant therapy? (Y/N)

☐ On anti-hypertensive therapy? (Y/N)

☐ On lipid-lowering therapy? (Y/N)

☐ Total cholesterol
☐ HDL cholesterol
(mmol/L to one decimal place [eg, 5.0] or mg/dL [eg, 200]: X = not available)

When completed, please keep copy in hospital notes and fax/post original to ACST Office, Dept of Cardiac & Vascular Sciences, St George's University of London, SW17 0RE, UK (fax +44 (0) 20 87 25 37 82)

ACST/R/1/1207

Appendix 3 – 1-month post-procedure form (page 1)

ACST-2 1-MONTH POST-PROCEDURE FORM: complete about 1 month after CEA/CAS

Name & address of doctor (or other person) completing this form (**PRINT**)

ACST 6-digit patient ID (eg 41-02-34) from randomisation or consent form, or **PRINT** patient's main names:

d d / m m / y y Date of birth (day/month/year)

Which procedure (CEA/CAS) was first attempted on the randomised artery? **Give details below**

A. Either: (1) CEA; Or: (2) CAS; Then: (1 or 2) procedural details

Either: (1) d d / m m / y y **Date of CEA** — **AND** Name of Surgeon, Hospital & City (**PRINT**)

Side of intervention? (L = Left, R = Right)

Patch used? Y = YES, N = NO

Shunt used? Y/N

Or: (2) d d / m m / y y **Date of CAS** — **AND** Name of Interventionalist, Hospital & City (**PRINT**)

Side of intervention? (L = Left, R = Right)

Type of stent? (S = Straight, T = Tapered)

Name of stent (**PRINT**)

Specialty of interventionalist? (S = Surgeon, R = Radiologist, C = Cardiologist, O = Other)

Cerebral protection device(s)? (N = none used, 1 = Distal balloon, 2 = Proximal occlusion, 3 = Filter)

Name(s) of CP device(s) (**PRINT**)

Then: (1 or 2) Procedural details (of CEA or of CAS)

Type of anaesthetic? (L = Local, G = General)

Anti-platelet drugs used? (A = Aspirin, C = Clopidogrel, O = Other, N = None); can enter 1 or 2 drugs

Hospital stay, to nearest whole day (99 = not yet discharged)

B. Post-procedure status

Ipsilateral cranial nerve damage from procedure? Y/N If **YES**, which cranial nerves? (*eg, XII*)

d d / m m / y y Date of post-procedure duplex Doppler

Comment:

Left side **Right side** % stenosis by this duplex Doppler (& any comment, if stenosis remains)

C. Other procedures done since randomisation

Any other procedures to this artery since randomised treatment? (CEA/CAS/N = None) If **YES** give date d d / m m / y y

Any procedures to contralateral artery since randomisation? (CEA/CAS/N = None) If **YES** give date d d / m m / y y

D. Events within 30 days after trial procedure (please answer ALL 3 questions)

**D1**	MI(s)?	Y/N	If **Yes**, give date	d d / m m / y y	and give details on next page →
**D2**	Stroke(s)?	Y/N	If **Yes**, give date	d d / m m / y y	and give details on next page →
**D3**	Death?	Y/N	If **Yes**, give date	d d / m m / y y	and give details on next page →

E. Current status (leave blank if dead) Date patient last seen d d m m y y

/ Systolic/diastolic blood pressure (mmHg)

Patient in hospital/nursing care now? Y/N (If **YES**, please **PRINT** address)

Currently on the following therapy? (Please answer **ALL 6** questions Y/N)

Aspirin — Clopidogrel — Other anti-platelet

Anti-coagulant — Anti-hypertensive — Lipid-lowering

When completed, please keep copy in hospital notes and fax/post original(s) to ACST Office, Dept of Cardiac & Vascular Sciences, St George's University of London, SW17 0RE, UK (fax +44 (0) 20 87 25 37 82)

ACST/FU1/1/1207

Appendix 3 – 1-month post-procedure form (page 2)

ACST-2 1-MONTH POST-PROCEDURE FORM: page 2 (leave page 2 completely blank unless a narrative is needed or there is a stroke, MI or death on page 1)

☐–☐–☐ ACST 6-digit patient ID (eg 41-02-34) from randomisation or consent form, or **PRINT** patient's main names: ....

If there was any peri- or post-procedural stroke, MI or death (within 1 month), describe briefly how the procedure went, its outcome and the clinical course and current status (with any relevant comments)

Details of major events within 1 month of the trial procedure (page 1, part D)

Time of event(s) after procedure (hours/days: please specify) ☐

Any comments on how the event(s) seemed to relate to the procedure?

D1 Myocardial infarction within 1 month

☐ Clinical symptoms? Y/N

☐ Definite ST-segment changes? Y/N

☐ Definite enzyme changes? Y/N

☐ Hospitalised for this event? Y/N

☐ If **YES**, length of stay (days, to the nearest whole day: 99 = not yet discharged)

Any comments (eg, on any additional infarcts)?

D2 Stroke within 1 month (If more than one, comment on all below)

☐ Laterality? (L = Left & R = Right carotid territory, O = Other; specify: ....)

☐ Type? (I = Ischaemic, H = Haemorrhagic, U = Unknown)

☐ Stroke confirmed by CT/MRI? Y/N (If **YES,** please send copy of report to ACST-2 office)

☐ Status from stroke at present (modified Rankin scale 0-6; see below)

☐ Hospitalised (or institutionalised) for this event? Y/N

☐ If **YES**, length of stay (days, to the nearest whole day: 99 = not yet discharged)

D3 Death within 1 month

Cause(s) of death

Any comments?

Any additional comments or information (as narrative)?

(eg, why allocated procedure not done; how procedure went; any further MIs or strokes; timing, location, nature & severity of all strokes etc.):

Modified Rankin Scale (NB If patient has stroke then dies of unrelated cause, describe stroke anyway)

0 No symptoms at all from the stroke.
1 No significant disability despite some symptoms: able to carry out usual duties and activities
2 Slight disability from the stroke: unable to carry out all previous activities but able to look after own affairs without assistance
3 Moderate disability from the stroke: requiring some help, but able to walk without assistance
4 Moderately severe disability from the stroke: unable to walk without assistance and unable to attend to own bodily needs without assistance
5 Severe disability from it: bedridden, incontinent and requiring constant nursing care and attention
6 Died directly or indirectly from the stroke

(You do not need to send this page if it is completely blank)

Appendix 4 – Patient annual follow-up letter

[Also available in various other languages on www.acst.org.uk]

ACST-2, a large international study comparing two types of stroke prevention

[PATIENT NAME]
[PATIENT ADDRESS, LINE 1]
[PATIENT ADDRESS, LINE 2]
[PATIENT ADDRESS, LINE 3]
[DATE]

Professor Alison Halliday, ACST office
Dept of Cardiac & Vascular Sciences
St George's University of London
London SW17 0RE, UK

[PATIENT NAME]
Study ID number: [PATIENT ID]

Dear [PATIENT],

On [DATE OF RANDOMISATION] you agreed to take part in an international study comparing two different procedures, CEA or CAS, to treat a narrowed artery in your neck in the hope of preventing stroke. I am now writing to find out how you have been since leaving hospital after having that procedure (CEA or CAS).

I need to know if you have had any kind of stroke since then and, if so, whether it still affects you. I hope that you will continue to be part of this study, and I would like to write to you again one year from now.

Please complete the short form and send it back in the envelope. If you cannot complete this form yourself, perhaps a friend, relative, carer or doctor can help. Thank you.

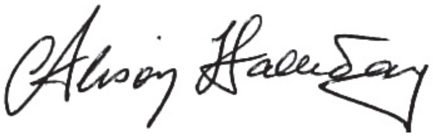

Dr. Alison Halliday, study director

Information you had before you joined the study
(and, on the next sheet, a copy of your previous agreement to join it)

Before you joined this study, your doctor had told you that you had a narrowing in one of the arteries in your neck that supplies blood to the brain, and that this narrowing could cause a stroke. Your doctor felt that it was time to deal with this narrowing. This study compares removing the narrowing by an operation (called carotid endarterectomy, CEA) or by keeping the inside of the artery open with a wire mesh tube (a procedure called carotid artery stenting, CAS), to see which is better. The findings of this study will help people like you in the future who have a narrowing in their carotid arteries.

You were told that we would want to write to you once a year for at least 5 years to ask you how you are. If we cannot contact you, we would like to ask your family doctor (or any friends or relatives you named) how you have been.

Further information about this study is available on www.acst.org.uk

Appendix 4 – Alternative follow-up letter (to family doctor, friend or relative)

[Also available in various other languages on www.acst.org.uk]

ACST-2, a large international study comparing two types of stroke prevention

[DOCTOR/FRIEND/RELATIVE NAME]
[ADDRESS, LINE 1]
[ADDRESS, LINE 2]
[ADDRESS, LINE 3]
[DATE]

Professor Alison Halliday, ACST office
Dept of Cardiac & Vascular Sciences
St George's University of London
London SW17 0RE, UK

[PATIENT NAME]
Study ID number: [PATIENT ID]

Dear [DOCTOR/FRIEND/RELATIVE NAME]

Some time ago, this patient agreed to take part in an international study of stroke prevention. I planned to write to them once a year to ask them how they have been, but this year I have had no answer. I am therefore writing to you to ask you whether you can help me. Copies of their agreement to join the study, and for me to try to contact you, are with this letter.

The main question is whether they have had any kind of stroke and, if so, whether it still affects them. If the questions cannot be answered, or if only some can be answered, please could you explain why on the second page of the questionnaire? Thank you.

Please complete the questionnaire and send it back. I hope that most or all of the questions can be answered, and I would like to write again one year from now and ask the same questions again if I cannot contact the patient directly. Thank you for your help.

Alison Halliday

Dr. Alison Halliday, study director

encl: Copy of letter recently sent by ACST office to the patient
Copy of annual questionnaire (which was for completion by the patient)

Further information about this study is available on www.acst.org.uk

Appendix 4 – Patient annual follow-up questionnaire (page 1)

[Also available in various other languages on www.acst.org.uk]

International study of stroke prevention procedures (Annual questionnaire; please complete BOTH pages)

Today's date d d / m m / y y day/month/year

Patient name (please **PRINT**)

Address (please **PRINT**), if different from that on the letter

Patient ID ☐–☐–☐ (from letter, to avoid mix-ups) (incl. tel & email, if known)

Please tick a box to say who filled out this form ☐ Patient ☐ Carer ☐ Friend/relative ☐ Other

We hope you have been well since leaving hospital after the neck artery procedure (CEA/CAS) you had when you first joined the study, but if not then please tell us.

1. Since you were last contacted d d m m y y, have you had a stroke?

Tick ☐ **Yes, or** ☐ **No.** If **YES**, what was the approximate date? d d / m m / y y

Which side of your body was affected? ☐ Left ☐ Right ☐ Neither side ☐ Both sides ☐ Don't know

Where were you treated? (can tick more than 1) ☐ Home ☐ Hospital/Clinic ☐ Other (eg, nursing home)

In total, how long were you in a hospital, clinic or nursing home because of it?

☐ days, or ☐ weeks, or ☐ months, or ☐ tick if still there

Do you know the name and address of a doctor who saw you (or of the hospital you went to)?

Name (PRINT):

Address (PRINT):

2. If you have had a stroke, how are you now? (Tick ONE box)

☐ No symptoms from the stroke
☐ Minor problems, but I can carry out everything I usually do
☐ A few problems from the stroke, but I can manage without help
☐ Problems from the stroke, I now need help with things
☐ Because of the stroke I now need help with most things

Anything else you'd like to tell us?

3. Since your first CEA/CAS, have you had any further neck artery procedures?

Tick box if **YES**:
☐ Operation (CEA) on my LEFT neck artery Date ☐/☐ (month/year, approx)
☐ Stent (CAS) in my LEFT neck artery Date ☐/☐ (month/year, approx)
☐ Operation (CEA) on my RIGHT neck artery Date ☐/☐ (month/year, approx)
☐ Stent (CEA) in my RIGHT neck artery Date ☐/☐ (month/year, approx)

If any answer is **YES**, did you have a stroke within the first month after the procedure? Yes or No ☐

4. Which medications do you take regularly?

Please **PRINT** the names of all prescription medicines you take regularly (ie, on most days), or state **NOT KNOWN**

continued over the page…

ACST/FU2/1/1207

Appendix 4 – Patient annual follow-up questionnaire (page 2)

International study of stroke prevention procedures
(Annual questionnaire; please complete BOTH pages)

5. Contact details

You gave us this information when you joined this study. We may need to contact one of these people if we cannot contact you when we write to you again next year.

Your family doctor	Your first friend or relative (1)	Your second friend or relative (2)

Please give new contact details, if they differ from those above
(thereby renewing your permission for us to contact them if necessary)

New name or contact details* for my family doctor (**PRINT**)	New name or contact details* for my first friend or relative (**PRINT**)	New name or contact details* for my second friend or relative (**PRINT**)

* ***(including tel. & email, if known)***

Thank you very much. Do you have any comments, further information or questions?

Name of person completing this form,signature and date

Patient ID ☐–☐–☐
(from letter, to avoid mix-ups)

Please put this form in the prepaid envelope provided (no stamp is needed),

OR post it in another envelope (with a stamp) to ACST office,
Dept of Cardiac & Vascular Sciences, St George's University of London, SW17 0RE, UK

This clinic is collaborating in a research project on how best to treat narrowed arteries in the neck

ACST patient information leaflet

If you have narrowed arteries and might be interested, please mention this to your doctor, or take an information leaflet

website:www.acst.org.uk

Memorandum of intent to collaborate in ACST-2: To be submitted prior to randomisation

Memorandum of intent to collaborate in ACST-2, incorporating the statement of local ethical approval for ACST-2

Name of Local Clinical Collaborator responsible for ethics approval: ____________________

Name and address of Institute or hospital: ____________________

____

____

ACST-2 is a long-term, large-scale randomised study comparing two standard procedural interventions for the treatment of patients with asymptomatic carotid artery stenosis ("Study"). The Study has ethics approval that was obtained by the above-named Local Clinical Collaborator at the above named Institute/hospital. All aspects of care at this Institute/hospital for any patient recruited into the Study shall at all times remain the responsibility of the Institute/hospital and its staff. The staff retain their right to disregard any aspect of the Study treatment allocation for that patient if, in their opinion, they consider it appropriate to do so. The Institute/hospital recognises that neither the Sponsor of ACST-2 (The University of Oxford, UK) nor St George's University of London, UK accept any liability for any aspect of the patient's treatment or its consequences.

The Institute/hospital and above-named Local Clinical Collaborator agree to conduct the Study in accordance with the principles of the Study protocol, but retain the right to withdraw from the Study or withdraw any patients from the Study at any time.

Signature on behalf of the University of Oxford, UK: ____________________

Signature of Local Clinical Collaborator: ____________________

Date signed: ____________________

Signature on behalf of the Institute/hospital: ____________________

Name (please PRINT): ____________________

Date signed: ____________________

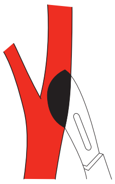

Asymptomatic Carotid Surgery Trial

ACST-2 PROTOCOL SUMMARY

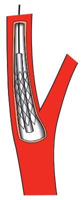

ELIGIBILITY (potential, then definite)

- **Potential eligibility:** Asymptomatic carotid stenosis that may well need procedural treatment with either carotid endarterectomy (CEA) or carotid artery stenting (CAS) The study can be mentioned and the ACST patient information leaflet given (or re-offered) either as soon as stenosis is found, or after further investigations, or both
- No symptoms from the stenosis (or none for some months), and no procedure previously performed on it. Any medical treatment (eg, statin, aspirin etc) already started; patient already recovered from any necessary coronary procedures (eg, CABG)
- **Definite eligibility:** MRA, CTA or other angiogram shows that CEA and CAS are both practicable: doctor **substantially uncertain** whether CEA or CAS is better (and sees no definite indication/contraindication for either*)

INFORMATION LEAFLET (can be re-offered) & consent

- Potentially and definitely eligible patients: mention the study and give (or re-offer) information leaflet (with an ACST doctor's name written onto the consent form) for the patient to read and discuss now and/or take away to consider and discuss later
- If the patient is then also **substantially uncertain** between CEA and CAS and is willing and eligible to join ACST, invite witnessed signature of the consent form
- Consent requires address of patient (for annual follow-up letter), of family doctor and of 1 or 2 friends or relatives (in case contact is lost). The information leaflet asks the patient to bring these along, but clinic staff may need to help the patient get them fully completed

ENTRY (by telephone randomisation)

- Complete at least part 1 of the randomisation form before telephoning to enter the patient, as these details are needed in the phone call. (The rest can be done later.)
- Ring the randomisation service +44 (0)18 65 76 56 15 to obtain the treatment allocation (CEA/CAS) and a 6-digit patient ID number
- Tell the patient which procedure (CEA/CAS) they have been allocated, and plan for that procedure to be done as soon as possible

PROCEDURE (performance, and 1-month follow-up)

- A collaborator with an approved Track Record for performing the allocated procedure does it, using their normal CEA/CAS techniques (& approved materials)
- Before discharge, schedule a follow-up about 1 month later for:
  - duplex ultrasound (to check carotid patency)
  - examination by neurologist/stroke physician (to assess & describe any peri-or post-operative stroke or MI)
- Complete and return the 1-month post-procedural form (stroke, MI or death); routine annual follow-up is then by letters to the patient from the ACST office

Randomisation: telephone +44 (0) 18 65 76 56 15
Website: www.acst.org.uk

*Reasons for not randomising are specified not by the protocol but by the responsible doctor, and might include

– **either** only a small likelihood of worthwhile benefit
  - Very low risk of stroke (eg, very minor stenosis)
  - Some major life-threatening disease (eg, advanced cancer)

– **or** a high risk of adverse events from CEA **or** from CAS
  - Access anatomically difficult either for CEA or for CAS
  - Unfit for surgery (eg, severe heart failure)
